# Mechanisms by which statins protect endothelial cells from radiation-induced injury in the carotid artery

**DOI:** 10.3389/fcvm.2023.1133315

**Published:** 2023-06-19

**Authors:** Karima Ait-Aissa, Linette N. Leng, Nathanial R. Lindsey, Xutong Guo, Denise Juhr, Olha M. Koval, Isabella M. Grumbach

**Affiliations:** ^1^ Abboud Cardiovascular Research Center, Department of Internal Medicine, Carver College of Medicine, University of Iowa, Iowa City, IA, United States; ^2^ Department of Biomedical Sciences, Dental College of Medicine, Lincoln Memorial University, Knoxville, TN, United States; ^3^ Free Radical and Radiation Biology Program, Department of Radiation Oncology, Carver College of Medicine, University of Iowa, Iowa City, IA, United States; ^4^ Iowa City VA Healthcare System, Iowa, IA, United States

**Keywords:** radiation therapy, carotid stenosis, endothelium, statin, mitochondria, prevention

## Abstract

**Background:**

The incidental use of statins during radiation therapy has been associated with a reduced long-term risk of developing atherosclerotic cardiovascular disease. However, the mechanisms by which statins protect the vasculature from irradiation injury remain poorly understood.

**Objectives:**

Identify the mechanisms by which the hydrophilic and lipophilic statins pravastatin and atorvastatin preserve endothelial function after irradiation.

**Methods:**

Cultured human coronary and umbilical vein endothelial cells irradiated with 4 Gy and mice subjected to 12 Gy head-and-neck irradiation were pretreated with statins and tested for endothelial dysfunction, nitric oxide production, oxidative stress, and various mitochondrial phenotypes at 24 and 240 h after irradiation.

**Results:**

Both pravastatin (hydrophilic) and atorvastatin (lipophilic) were sufficient to prevent the loss of endothelium-dependent relaxation of arteries after head-and-neck irradiation, preserve the production of nitric oxide by endothelial cells, and suppress the cytosolic reactive oxidative stress associated with irradiation. However, only pravastatin inhibited irradiation-induced production of mitochondrial superoxide; damage to the mitochondrial DNA; loss of electron transport chain activity; and expression of inflammatory markers.

**Conclusions:**

Our findings reveal some mechanistic underpinnings of the vasoprotective effects of statins after irradiation. Whereas both pravastatin and atorvastatin can shield from endothelial dysfunction after irradiation, pravastatin additionally suppresses mitochondrial injury and inflammatory responses involving mitochondria. Clinical follow-up studies will be necessary to determine whether hydrophilic statins are more effective than their lipophilic counterparts in reducing the risk of cardiovascular disease in patients undergoing radiation therapy.

## Graphical Abstract

Central Illustration: The effects of pravastatin and atorvastatin pretreatment in radiation-induced mitochondrial and endothelial damage. While pravastatin treatment prevents short- and long-term cytosolic and mitochondrial oxidative stress, inflammation and endothelial injury, atorvastatin treatment does only protect against the cytosolic oxidative stress at short-term and does not counteract radiation-induced mitochondrial damage leading to inflammation.

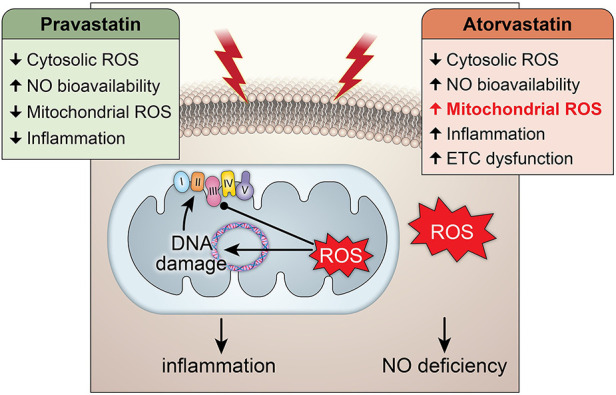

## Introduction

Radiation therapy (RT) strongly increases the risk of developing atherosclerotic vascular disease in coronary and peripheral arteries (1–4). In head-and-neck cancers, RT is an important and potentially curative modality and the preferred treatment in localized disease. For more advanced disease, it is combined with chemotherapy as a definitive organ function-preserving approach, or after surgery as an adjuvant therapy. Because of the proximity of the carotid artery to the lymphatic structures that are targeted by RT, the risk of developing significant carotid stenosis is elevated in this population (5–8). The rate of progression of carotid artery stenosis to >50% was 15.4% per year in patients who had undergone RT vs. 4.8% (an ∼3-fold difference) in patients who had not received RT but were matched for the baseline severity of carotid artery stenosis (9). This discrepancy increased with progression to more severe stenosis, with the risk of progression to >70% stenosis reported to be 7-fold higher in patients who had undergone RT for head-and-neck cancer vs. those who had not (10). The incidence of stroke was also higher in patients who had even limited neck RT vs. patients treated with surgery alone (11–13).

It may be possible to prevent irradiation-induced carotid artery stenosis by administering specific mitigators during RT. However, this would require a more complete understanding of the pathogenesis of irradiation-induced vascular injury. In the absence of these data, the efficacy of other medications known to lower the risk of atherosclerotic cardiovascular disease and used incidentally in cancer survivors has been assessed. These medications include aspirin, colchicine, and statins [hydroxymethyl-glutaryl coenzyme A (HMG-CoA) reductase inhibitors] (14). The studies revealed that the incidental use of statins at the time of, or after, RT for head-and-neck cancer is associated with a lower risk of stroke (15, 16). Although it is commonly accepted that statins (17) have antioxidant effects, the specific molecular pathways that account for the protection from radiation injury remain to be established. Mitochondria are a major source of intracellular ROS production and damaged by RT (18–21). One major cause of RT-induced vascular disease is the injury of endothelial cells (ECs), which are highly sensitive to radiation (22). Such injury leads to significant impairment of endothelium-dependent dilation of human carotid arteries at 4–6 weeks after RT (23). Thus, endothelial dysfunction, defined as impaired endothelium-dependent dilation in response to nitric oxide (NO), can be regarded as a clinically relevant early indicator of carotid injury after RT (24).

The aim of this study was to ascertain whether statins protect from endothelial dysfunction after RT by preventing the production of mitochondrial reactive oxygen species (ROS) and its downstream sequelae, loss of mitochondrial membrane potential and DNA damage. We also hypothesized that two statins with distinct chemical properties, pravastatin (hydrophilic) and atorvastatin (lipophilic) have similar effects. For this purpose, we tested C57B/6 mice at 24 and 240 h after RT and dissected the underlying molecular mechanisms in cultured human coronary artery and umbilical vein endothelial cells at the same time points.

## Materials and methods

### Mice

All experimental procedures were approved by the Institutional Animal Care and Use Committees of both the University of Iowa and the Iowa City VA Health Care System and complied with the standards of the Institute of Laboratory Animal Resources, National Academy of Sciences. Fifteen males and 15 females C57BL/6J mice were obtained from Jackson Laboratories (#000664). All mice were between 12 and 16 weeks of age at the time of treatment (5 mice per group of treatment).

#### Statin treatment of mice

Pravastatin (*n* = 10) and atorvastatin (*n* = 10) were given orally in drinking water to provide doses of 30 and 3 mg/kg/day, respectively (25, 26). The treatment started 72 h before RT and was continued for up to 10 days after RT. Mice in the control group (*n* = 10) received vehicle only (filtered tap water) according to the same schedule. The doses and the treatment regimen were chosen based on the range reported as exerting effects on vascular function in animal models (27, 28).

#### Radiation exposure of mice

Anesthetized mice were irradiated using the XStrahl Small Animal Radiation Research Platform (SARRP) with a single anterior–posterior beam and a beam quality of 0.67 mm Cu. The dose rate used was 3.6 Gy/min and was calibrated at 2 cm depth in water, in accordance with the AAPM TG-61 protocol. A dose of 12 Gy x-rays, which equates to EQD2 dose of 36 Gy (α/β of 3), was delivered to the whole brain in a single session. Simulation was performed with computed tomography. The accuracy of dosimetry by the SARRP was ensured by quarterly measurements of the ion chamber by a medical physicist (29).

### Measurement of vascular reactivity

Arterial rings were prepared from the carotid and second-branch mesenteric resistance arteries (MRAs) and their isometric tension was measured after they were mounted in a small vessel dual chamber myograph. MRAs were used as control arteries (from a vascular bed outside the radiation field). Following equilibration in Krebs solution bubbled with CO_2_ at 37°C and at pH 7.4 for 30 min, the rings were stretched to their optimal physiological lumen diameter for 1 h to develop active tension. The rings were then pre-constricted with phenylephrine (PE, 3 × 10^−5^ M), after which they were treated with acetylcholine (ACh, 10^−8^–3 × 10^−5^ M) and sodium nitroprusside (SNP, 10^−8^–3 × 10^−5^ M) and cumulative concentration-response curves were generated. Data from male and female mice were initially analyzed separately. The results reported in this manuscript were combined because no difference was seen between the two groups.

### Endothelial-cell cultures and treatments

Primary HCAECs and HUVECs were grown in endothelial cell medium (ECM, #1001, ScienCell) supplemented with 5% fetal bovine serum (FBS), 1% endothelial growth supplements, and 1% penicillin/streptomycin at 37°C and 5% CO_2_ and used at passages 3–5.

#### Statin treatment

ECs were treated with 10 μM pravastatin or 5 μM atorvastatin in DMSO for 18 h before irradiation. Control cells were treated with DMSO only.

#### Irradiation of cultured ECs

Once ECs were 80% confluent, they were exposed to γ-rays (4 Gy). Ionizing radiation was delivered at 1.29 Gy/min using a cesium-137 γ-ray source in the Radiation and Free Radical Research Core of the University of Iowa. At 24 and 240 h following irradiation, ECs were collected for analyses of the levels of mRNA and proteins, as well as for imaging.

### Measurement of cellular ROS production

Cellular ROS production was measured in cultured HCAECs using the chloromethyl derivative of 7′-dichlorodihydrofluorescein diacetate, acetyl ester (CM-H_2_DCFDA) (5 μM) (30). Cells were loaded with CM-H_2_DCFDA (5 μM) for 45 min at 37°C. The cells were then imaged at an excitation wavelength of 495 nm and an emission wavelength of 520 nm, and the images were analyzed using NIH ImageJ. All images were taken using the same settings. Specifically, CM-H_2_DCFDA fluorescence signals were traced per cell and data are presented as integrated density. Samples treated with the superoxide scavenger 4-hydroxy-2,2,6,6-tetramethylpiperidine-1-oxyl (TEMPO) prior to IR, and samples treated with H_2_O_2_ only, served as negative and positive controls, respectively.

### Measurement of mitochondrial ROS production

Mitochondrial ROS (mitoROS) production was measured in cultured HCAECs using the dihydroethidium derivative mitoSOX red (5 μM) (31). Cells were loaded with mitoSOX (5 μM) and MitoTracker Green FM (1 μM) (32) for 20 min at 37°C. The cells were then imaged using a NIKON microscope and analyzed using NIH ImageJ. All images were taken using the same settings. Specifically, mitoSOX and the MitoTracker Green FM signals were traced per cell and the fluorescence intensity of mitoSOX was normalized to that of MitoTracker Green FM. Data are presented as the ratio of the integrated density mitoSOX signal to MitoTracker Green FM signal.

### Measurement of mitochondrial membrane potential

The mitochondrial membrane potential was measured in HCAECs using tetramethylrhodamine methyl ester (TMRM, cat # T668, Molecular Probes). Each well was seeded with 50,000 cells, and these were grown in phenol-free endothelial cell growth medium. Cells were rinsed once with PBS, loaded with TMRM (100 nM) for 20 min at 37°C, and then rinsed once with PBS. Images were acquired at an excitation wavelength of 544 nm and an emission wavelength of 570 nm, using a Nikon Eclipse Ti2 microscope (objective 40X). Images were analyzed using the NS Elements software (Nikon).

### Quantification of nitric oxide

NO levels in HCAECs were measured using the fluorescent probe DAF2-DA (Sigma) (33). Once the cells achieved confluency, they were exposed to DAF2-DA (5 µM) for 10 min. They were then imaged under a fluorescence microscope. PDGF (20 ng/ml) + glutamine (1 µM) was used to stimulate NO production and continuous imaging was performed for 10 min following treatment. The amount of NO produced is expressed as fluorescence intensity normalized to baseline. L-NNA (competitive inhibitor of NO synthase) and SNP were used as negative and positive controls, respectively.

### Assessment of damage to _mt_DNA

Quantitative PCR (QPCR) was used to assay _mt_DNA damage as described previously (34). Briefly, total DNA (20 ng) was amplified using the Platinum PCR Super Mix (Invitrogen, USA). Specific primer sets were used to amplify both a long fragment of the _mt_DNA (8.9-kb) (to determine its integrity) and a short fragment (139-bp). The shorter fragment was amplified so that its integrity could be determined, and the longer fragment was amplified so that changes in _mt_DNA copy number could be monitored and for use in normalizing the data obtained from amplification of the longer fragment. Ratios of relative amplification were calculated to compare _mt_DNA damage in irradiated ECs to that in non-irradiated ECs; these values were used to express the number of lesions present in DNA, assuming a Poisson distribution, as previously described (34).

### Quantitative real-time PCR

Total RNA extracted from cultured cells was used in real-time PCR performed in a ViiA 7 Real-Time PCR System (Applied Biosystems, Foster City, CA). Primers were designed by Integrated DNA Technologies to amplify the following genes: NFkB-p50, NFkB-p65, TNFα, cytochrome c oxidase I (mt-COI), NADH-ubiquinone oxidoreductase chain 1 (mt-ND1), NADH dehydrogenase [ubiquinone] 1 alpha subcomplex subunit 1 (NDUF1), cytochrome c oxidase 11 (COX11) and ribosomal 18S (internal control).

### Assessment of activity of ETC complex 1

The activity of ETC complex 1 in HCAEC lysates was measured using the MitoCheck® Complex I Activity Assay Kit (#700930, Cayman chemical, Ann Arbor, MI, USA) according to the manufacturer's instructions.

### Statistical analysis

In all cell culture experiments, we employed a comprehensive approach that included careful experimental design, randomization, and rigorous data normalization techniques. Sell culture experiments were performed in different batches of cells and included multiple controlsto account for systematic variations. Additionally, we implemented strategies to address technical variability, such as using standard reference samples or including technical replicates within and across batches.

Data are expressed as mean ± standard error of the mean (SEM) and were analyzed using the GraphPad Prism 9.0 software. All data sets were analyzed for normality and equal variance. The Kruskal-Wallis and Dunn's *post hoc* tests were used for data sets where a normal distribution could not be assumed. The two-tailed unpaired Student's *t*-test and one-way ANOVA, followed by Tukey's multiple comparison test, were used for data sets with a normal distribution. Two-way ANOVA followed by Tukey's multiple comparison test was used for grouped data sets. A *p*-value < 0.05 was considered significant.

## Results

### Pretreatment with pravastatin and atorvastatin protects endothelial function equally

**Figure 1:**
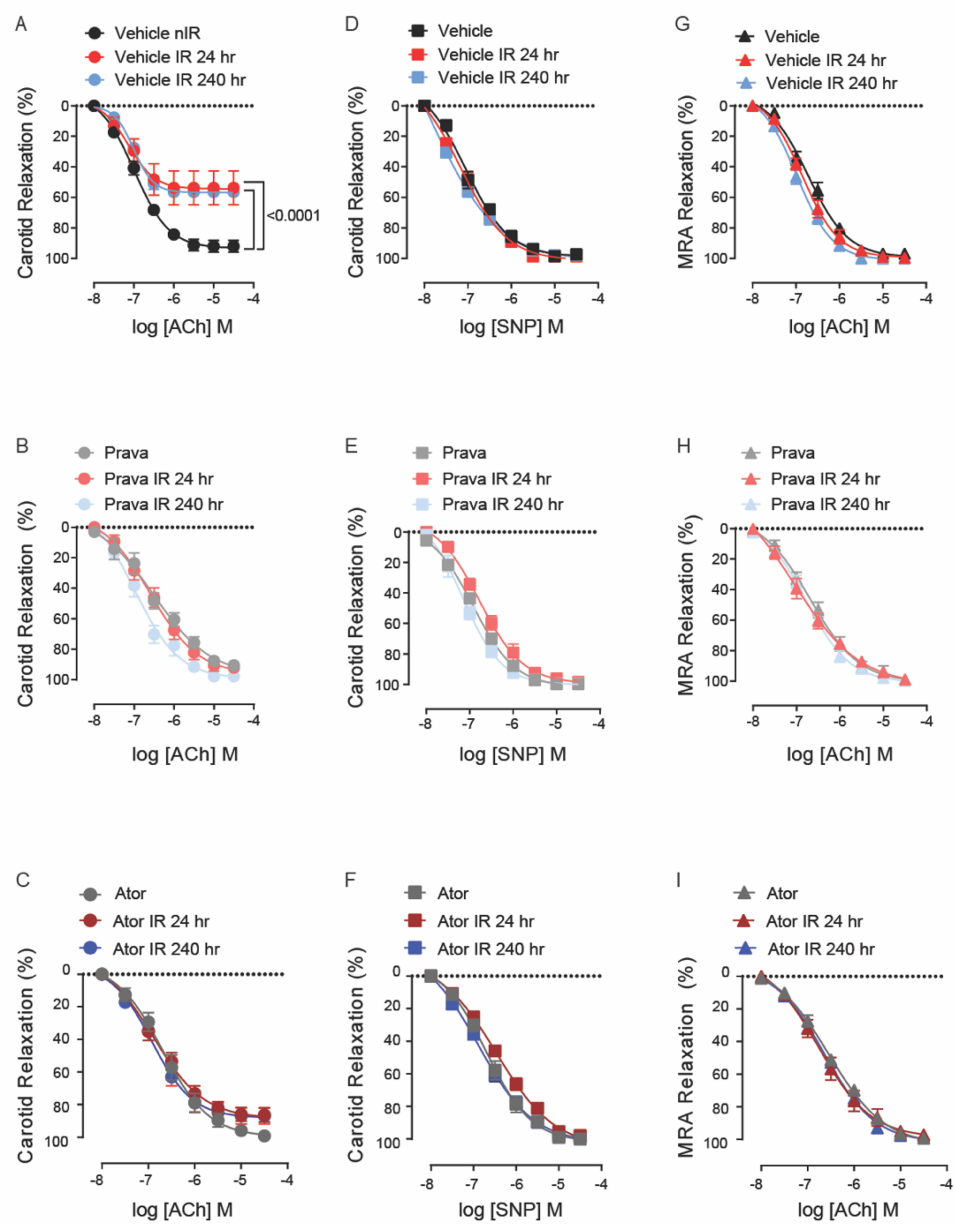
Pravastatin and atorvastatin preserve endothelial function in vivo following head-and-neck IR. **(A–C)** Effects of statins on endothelium-dependent relaxation of the carotid artery in response to acetylcholine (ACh). C57BL/6J mice were treated with **(A)** vehicle, **(B)** pravastatin (Prava), or **(C)** atorvastatin (Ator) after head-and-neck irradiation (12Gy) or sham treatment, and relaxation was tested at 24 and 240 hr after irradiation. **(D–F)** Effects of statins on endothelium-independent relaxation of the carotid artery in response to sodium nitroprusside (SNP), in mice treated as in A, B, and C, respectively. **(G–I)** Effects of statins on endothelium-dependent relaxation of mesenteric resistance arteries (MRAs), in mice treated as in A, B, and C, respectively. n=5 mice per group. *p* values were determined using repeated measures 2-way ANOVA followed by Tukey's post-Hoc test.

To assess the effect of pravastatin and atorvastatin on vascular reactivity following irradiation, we measured relaxation of the common carotid artery in response to treatment with acetylcholine (Ach, endothelium-dependent) and sodium nitroprusside (SNP, endothelium-independent). C57BL/6J mice were treated with either pravastatin, atorvastatin, or vehicle only (control) and then subjected to irradiation (12 Gy x-ray) of the head and neck. At 24 and 240 h, relaxation in response to ACh was significantly less pronounced in the carotid arteries of irradiation- compared to sham-treated mice (*p* < 0.0001; Figure 1A). Both pravastatin and atorvastatin preserved endothelium-dependent relaxation at 24 and 240 h post-irradiation, and did so to a similar extent (Figures 1B,C). Notably, endothelium-independent relaxation of the carotid artery (i.e., in response to SNP) was not affected by irradiation and was similar in all groups (Figures 1D–F). This was also the case for relaxation in response to ACh in mesenteric resistance arteries (MRAs) (Figures 1G–I), which lie outside the radiation field.

The reported effects of RT include increased levels of cellular ROS, and this has been shown to impair both NO bioavailability and endothelial function (35, 36). We tested the abilities of pravastatin and atorvastatin to reduce ROS levels and preserve NO production in HCAECs following irradiation. In pilot studies, we compared the effects of pravastatin and atorvastatin on EC viability by cell counts and MTT assays. We tested 5 and 10 μM of each compound because these doses were used in previous studies (37–41). Whereas pravastatin had no effect at either concentration, the 10-μM dose of atorvastatin decreased cell counts compared to vehicle and 5 μM atorvastatin (Supplementary Figures S1A,B) or viability compared to vehicle and pravastatin treatment in MTT assays (Supplementary Figure S1C). Thus, for our *in vitro* studies, we treated cells with 10 μM pravastatin or 5 μM atorvastatin.

**Figure 2:**
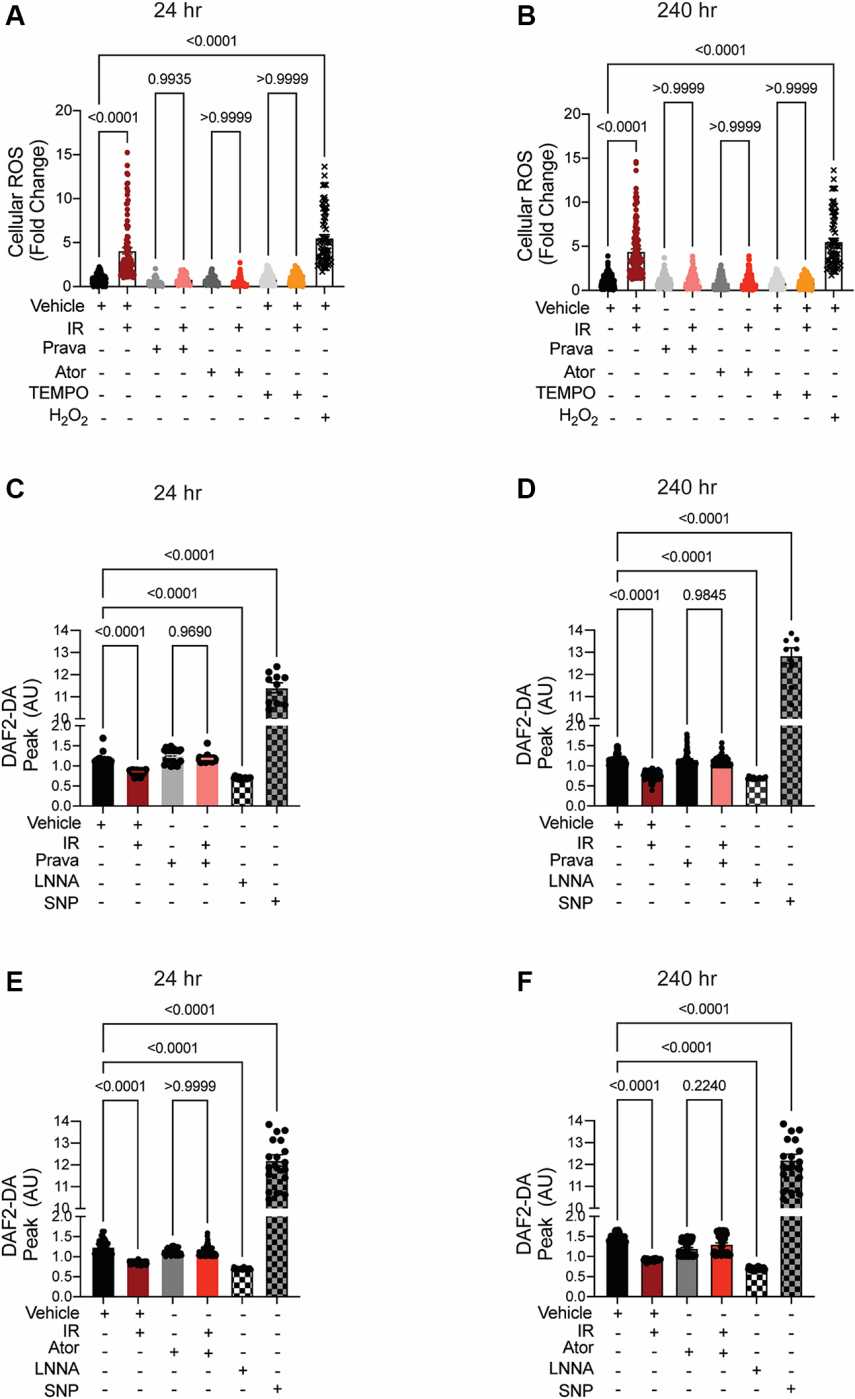
When administered before IR *in vitro*, pravastatin and atorvastatin prevent IR-induced ROS production and loss of NO production. All panels compare HCAECs subjected to irradiation (4 Gy). **(A,B)** Change in cellular ROS levels, as determined by CM-H_2_DCFDA fluorescence at **(A)** 24 and **(B)** 240 h after IR, in cells treated with vehicle, pravastatin (Prava), or atorvastatin (Ator) starting at 18 h before irradiation (4 Gy). HCAECs treated with TEMPO or H_2_O_2_ served as negative and positive controls, respectively. **(C–F)** NO production in cells pretreated with pravastatin **(C,D)** or atorvastatin **(E,F)** and in response to PDGF (20 ng/ml), as determined by DAF2-DA fluorescence. NO levels are normalized to baseline (i.e., levels before PDGF addition) and plotted as fold-change relative to untreated cells. HCAECs treated with L-NNA and SNP served as controls. Images were taken at **(C,E)** 24 and **(D,F)** 240 h after irradiation. *n* = 4 independent experiments. *p* values by Kruskal-Wallis test.

In irradiated HCAECs, at both 24 and 240 h the levels of intracellular ROS detected by CM-H_2_DCFDA were significantly higher than in non-irradiated counterparts (*p* < 0.0001; Figures 2A,B). Pretreatment with either pravastatin or atorvastatin completely blocked the increases in ROS levels at both time points. Consistent with the concept that increases in intracellular ROS lead to reduced bioavailability of NO in vascular beds (35), NO production by HCAECs after irradiation was significantly reduced at both time points, and this effect was abolished by pretreatment with statins (*p* < 0.0001; Figures 2C–F).

These data indicate that pravastatin and atorvastatin have comparable abilities to prevent the irradiation-induced cellular ROS production that leads to a deficiency in NO production, and ultimately to endothelial dysfunction, in arteries within the field of radiation.

### Pretreatment with pravastatin, but not atorvastatin, protects against mitochondrial injury after irradiation

**Figure 3:**
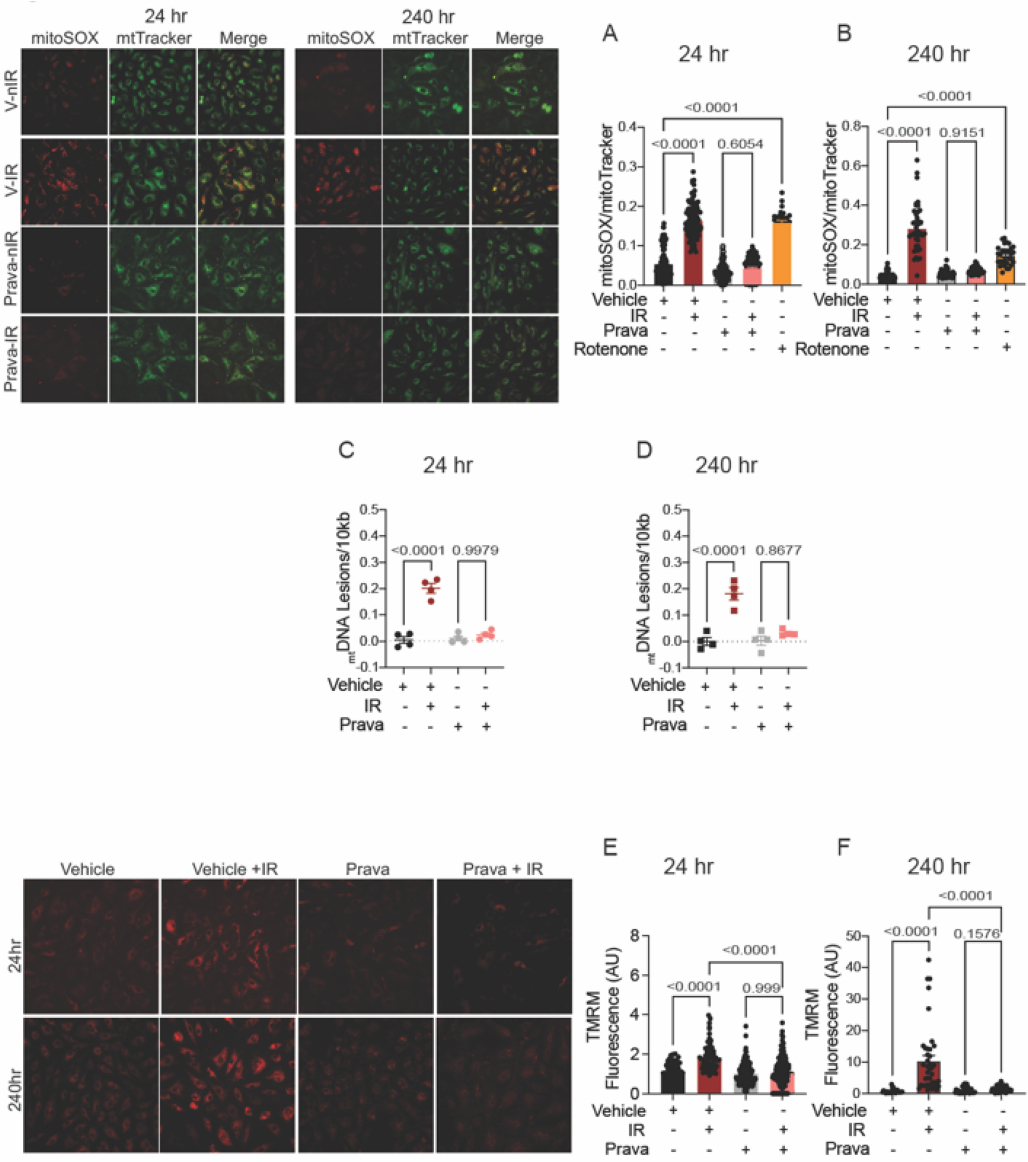
Pravastatin protects against IR-induced mitochondrial damage or hyperpolarization *in vitro*. All panels compare cells subjected to irradiation (4 Gy) after pretreatment with pravastatin (Prava, 10 mM) starting at 18 hr before irradiation. **(A,B)** Representative images and signal integrated density of mitoSOX fluorescence normalized to mitoTracker fluorescence in HCAECs at **(A)** 24 and **(B)** 240 hr after IR. **(C,D)** mtDNA lesions in DNA extracted from HUVECs at **(C)** 24 hr and **(D)** 240 hr after IR. **(E,F)** Representative images and integrated density of mitochondrial membrane potential in HCAECs, as determined by TMRM fluorescence, at **(E)** 24 hr and **(F)** 240 hr after irradiation. Analysis per cell, *n* = 4 independent experiments. *p* values were determined by Kruskal-Wallis test.

Irradiation leads to increases in the levels of mitoROS, i.e., greater mitochondrial injury (42). Here, we sought to test whether pravastatin and atorvastatin provide protection against specifically mitochondrial injury after irradiation by measuring mitoROS levels using mitoSOX (levels were normalized to mitochondrial mass, as determined by MitoTracker). As anticipated, at both 24 and 240 h post-irradiation, mitoROS production was elevated (*p* < 0.0001; Figures 3A,B), and this effect was abolished by treatment with pravastatin (*p* = 0.6054). Treatment with rotenone (positive control) promoted mitoROS production in the absence of irradiation (*p* < 0.0001; Figures 3A,B). One outcome of irradiation is damage to the mitochondrial DNA (_mt_DNA). This DNA is particularly susceptible to radiation in part because it lacks histones and several DNA repair mechanisms, but also because it is located near components of the electron transport chain (ETC), the source of ROS in mitochondria. Thus, we measured lesions in _mt_DNA isolated from HUVECs that had been subjected to irradiation, with or without pravastatin pretreatment. In vehicle-treated HUVECs, the occurrence of _mt_DNA damage following irradiation was significantly higher than in untreated controls (*p* < 0.0001 compared to non-IR; Figures 3C,D). Consistent with our findings for mitoROS, pravastatin reduced _mt_DNA damage after irradiation (at 24 h: *p* = 0.9979 and at 240 h: *p* = 0.8677 compared to non-IR pravastatin; Figures 3C,D). Next, we measured the effects of statins on the mitochondrial membrane potential (Δψ_mt_). Following irradiation, HUVECs showed significantly elevated Δψ_mt_, and this was attenuated by pravastatin pretreatment (*p* < 0.0001 compared to irradiated vehicle; Figures 3E,F).

**Figure 4:**
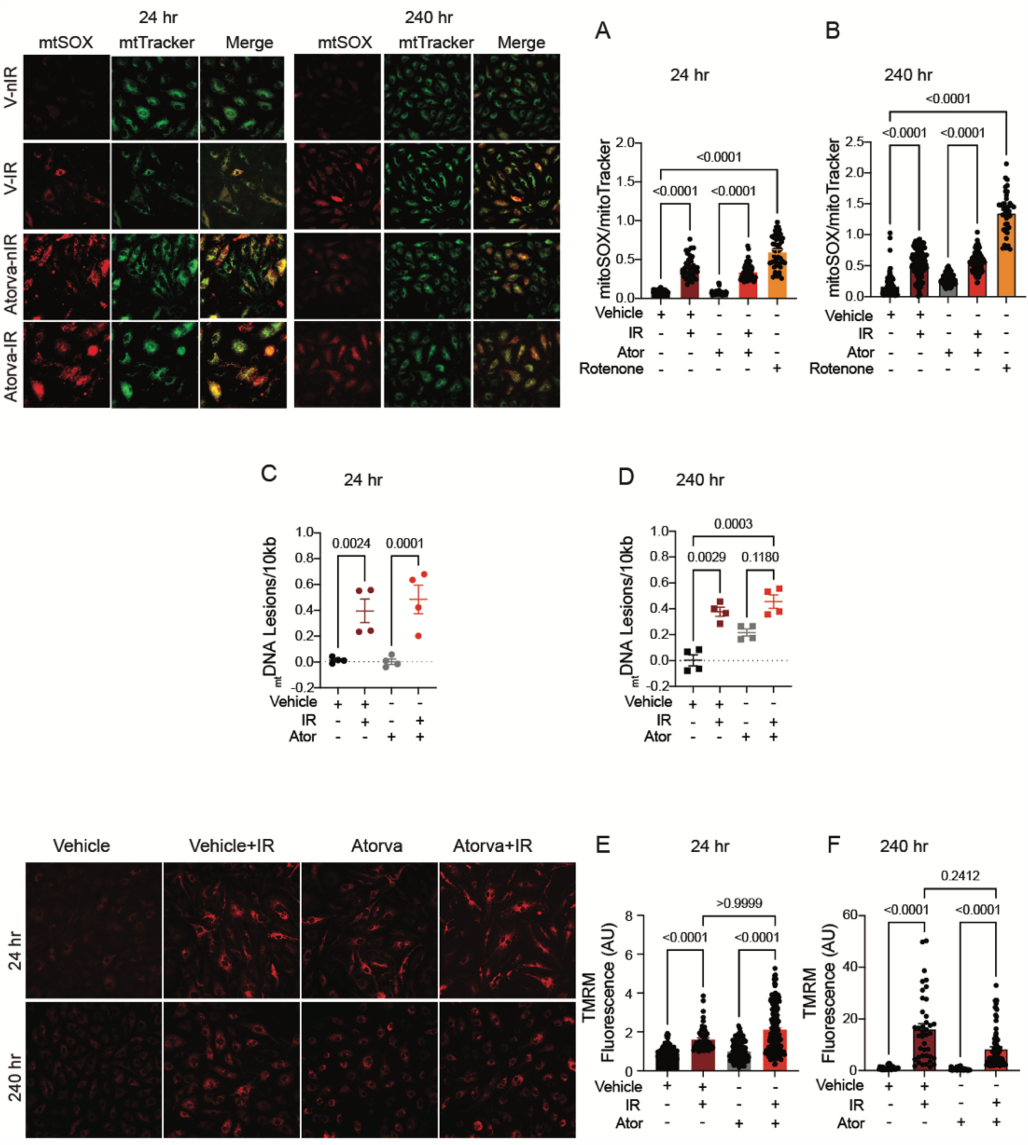
Atorvastatin does not protect against IR-induced mitochondrial damage or hyperpolarization *in vitro*. All panels compare HCAECs subjected to irradiation (4 Gy) after pretreatment with atorvastatin (Ator, 5 mM) or vehicle. Parameters assessed are: **(A,B)** Representative images and signal integrated density of MitoSOX fluorescence normalized to MitoTracker fluorescence at **(A)** 24 and **(B)** 240 hr after irradiation, in cells treated with atorvastatin (5 mM) or vehicle starting 18 hr before irradiation. **(C,D)** Damage to mtDNA as assessed by PCR assay. mtDNA lesions at **(C)** 24 and **(D)** 240 hr after irradiation, in HUVECs treated with atorvastatin or vehicle starting 18 hr before IR. **(E,F)** Representative images and integrated density of mitochondrial membrane potential, as determined by TMRM fluorescence, at **(E)** 24 and **(F)** 240 hr after irradiation. Analysis per cell, *n* = 4 independent experiments, *p* values by Kruskal-Wallis test.

Unexpectedly, pretreatment with atorvastatin had no protective effect on mitoROS production at either 24 or 240 h (*p* < 0.0001 compared to non-IR atorvastatin; Figures 4A,B). Similarly, pretreatment with atorvastatin did not prevent _mt_DNA damage following irradiation at either time point (*p* < 0.0001 (24 h) and *p* = 0.0003 (240 h) compared to non-IR vehicle; Figures 4C,D). Moreover, the increase in Δψ_mt_ after irradiation was similar in atorvastatin- and in vehicle-treated HUVECs (*p* < 0.0001 compared to non-IR atorvastatin; Figures 4E,F). These findings reveal that, unlike pravastatin, atorvastatin does not preserve mitochondrial function post-irradiation.

### Pretreatment with pravastatin, but not atorvastatin, blocks chronic inflammatory signaling post-irradiation

**Figure 5:**
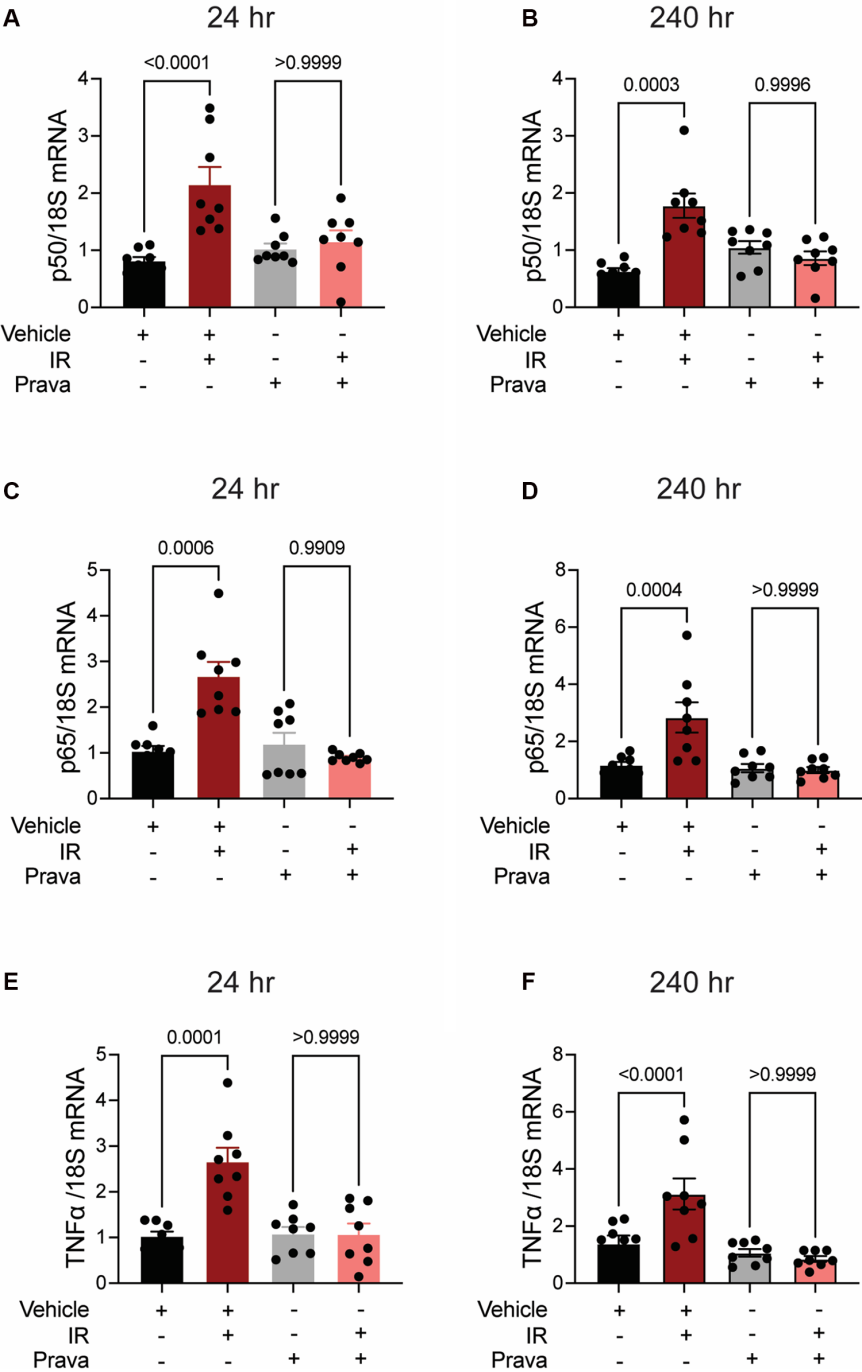
Pravastatin prevents IR-induced inflammatory signaling. All panels compare HCAECs subjected to irradiation (4 Gy) after pretreatment with pravastatin (Prava, 10 μM, overnight) or vehicle. **(A,B)** Quantitative (q)RT-PCR for NFκB-p50 at **(A)** 24 and **(B)** 240 h after irradiation. **(C,D)** qRT-PCR for NFκB-p65 at **(C)** 24 and **(D)** 240 h after irradiation. **(E,F)** qRT-PCR for TNF*α* at **(E)** 24 and **(F)** 240 h after irradiation. *p* values by Kruskal-Wallis test.

**Figure 6:**
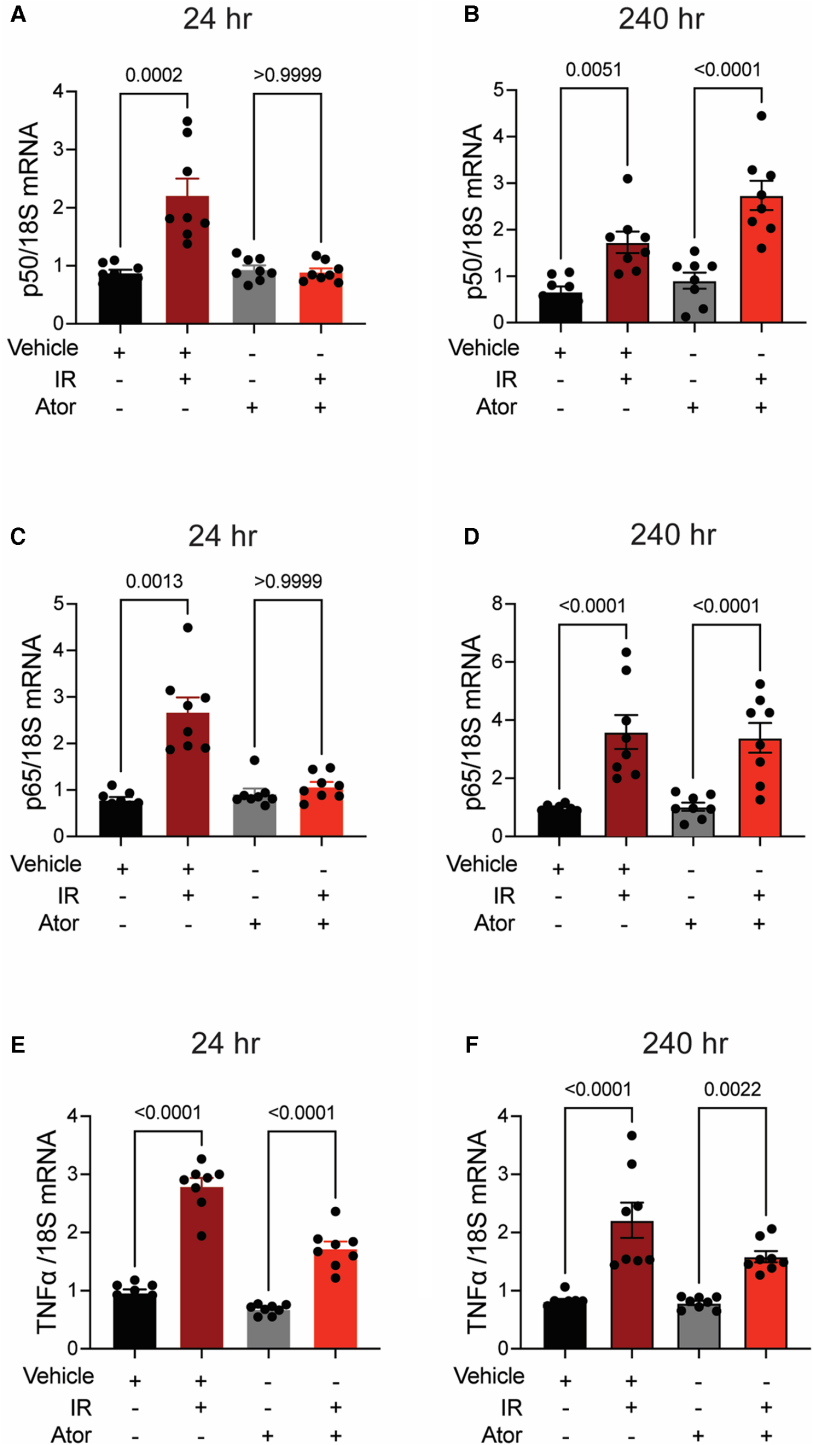
Atorvastatin does not prevent IR-induced inflammatory signaling. All panels compare HCAECs subjected to irradiation (4 Gy) after pretreatment with atorvastatin (Ator, 5 μM, overnight) or vehicle. **(A,B)** Quantitative (q)RT-PCR for NFκB-p50 at **(A)** 24 and **(B)** 240 h after irradiation. **(C,D)** qRT-PCR for NFκB-p65 at **(C)** 24 and **(D)** 240 h after irradiation. **(E-F)** qRT-PCR for TNFα at **(E)** 24 and **(F)** 240 h after irradiation. *p* values by Kruskal-Wallis test.

Next, we investigated pro-inflammatory pathways that are activated downstream by mitoROS after irradiation. We tested whether irradiation-induced increases in the expression of TNFα and NFκB are abolished by statin pretreatment (43–45). In vehicle-treated HCAECs, levels of the NFκB-p50 (*p* < 0.0001; *p* = 0.0003), NFκB-p65 (*p* = 0.0006; *p* = 0.0004), and TNF*α* (*p* = 0.0001; *p* < 0.0001) mRNAs were elevated at 24 and 240 h respectively. Pravastatin treatment blocked the expression of these markers at both 24 and 240 h following irradiation (Figures 5A–F). In contrast, although atorvastatin treatment inhibited upregulation of the NFκB-p50 (*p* = 0.0002; Figure 6A) and NFκB-p65 (*p* = 0.0013; Figure 6C) mRNAs at 24 h post-irradiation, it failed to block the expression of either NFκB or TNFα at 240 h (*p* < 0.0001, Figures 6B,D,F; *p* < 0.0001, *p* = 0.0022). In the case of TNFα, atorvastatin did not affect expression at either timepoint (*p* < 0.0001, Figures 6E,F). The protein levels of NFκB-p65 was also assessed and the results exhibited similar patterns as the mRNA results (Supplemental Figure S2). These findings suggest that TNFα expression may be driven by NFκB upregulation and lead to a sustained excess of mitoROS after RT.

### The effects of pretreatment with pravastatin and atorvastatin on mitochondrial DNA transcription and ETC function after irradiation differ

Next, we investigated the mechanisms by which pravastatin protects against sustained mitoROS production following irradiation. We reasoned that excess ROS are by-products of dysregulation of the activity of the ETC. Because damage to _mt_DNA occurs during irradiation, and it is sustained at 240 h whereas damage to nuclear DNA (_nuc_DNA) is not (46, 47), we hypothesized that the transcription of mitochondrial (but not nuclear) subunits of ETC complexes is impaired after irradiation and leads to loss of ETC activity and increased mitoROS production.

**Figure 7:**
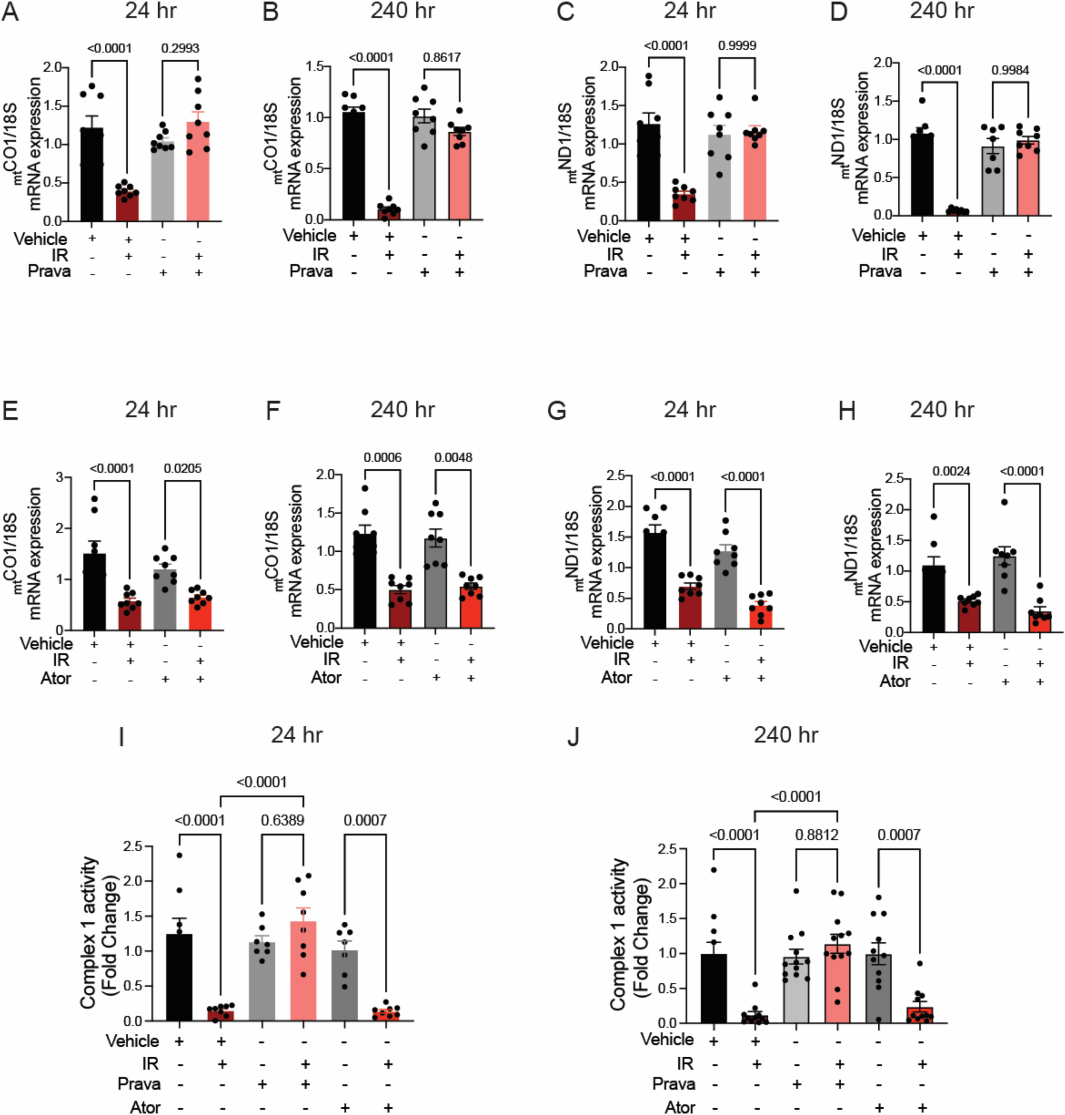
Pravastatin, but not atorvastatin, prevents irradiation-induced reduction of mtDNA transcription and ETC activity. All panels compare HCAECs subjected to irradiation (4 Gy) after pretreatment with atorvastatin (Ator, 5mM, overnight), pravastatin (Prava, 10mM, overnight) or vehicle. **(A–D)** Effects of pretreatment with pravastatin (Prava, 10mM, overnight) on transcriptional activity. **(A,B)** Quantitative (q)RT-PCR for cytochrome c oxidase I (MT-COI) at **(A)** 24 and **(B)** 240 hr after irradiation. **(C–D)** qRT-PCR for NADH-ubiquinone oxidoreductase chain 1 (MT-ND1) at (C) 24 and **(D)** 240 hr after irradiation. **(E–H)** Effects of pretreatment with atorvastatin (Ator, 5 mM, overnight) on transcriptional activity. **(E,F)** qRT-PCR for MT-COI at **(E)** 24 and **(F)** 240 hr after irradiation. **(G,H)** qRT-PCR for MT-ND1 at **(G)** 24 and **(H)** 240 hr after irradiation. **(I,J)** Activity of ETC complex 1, as assessed by fluorometric assay at **(I)** 24 and **(J)** 240 hr after irradiation. *p* values by Kruskal-Wallis test.

To test this hypothesis, we performed qRT-PCR for the ETC subunits _mt_COI and _mt_ND1, which are transcribed from the _mt_DNA. Irradiation significantly reduced transcript levels at 24 and 240 h (Figures 7A–H). Whereas preincubation with pravastatin prevented this effect (Figures 7A–D), preincubation with atorvastatin did not (_mt_COI 24 h *p* = 0.0205, 240 h *p* = 0.0048 and _mt_ND1 24 h *p* = 0.0205, 240 h *p* = 0.0048 compared to non-IR atorvastatin) (Figures 7E–H). In contrast, the transcription of two subunits encoded by _nuc_DNA, NDUF1 and COX11, was unaffected by irradiation (Supplementary Figure S3). This dissociation of transcriptional effects, based on whether ETC subunits are encoded by nuclear or mitochondrial DNAs, is consistent with ETC activity being impaired after irradiation. Indeed, activity of ETC complex 1 was reduced at 24 h (*p* < 0.0001 compared to non-IR vehicle), and this effect was blocked by pravastatin treatment (*p* < 0.0001 compared to IR vehicle; Figures 7I,J). However, as in the case of mitoROS production and _mt_DNA production, atorvastatin failed to prevent impairment of the activity of ETC complex 1 (*p* = 0.0007 compared to non-IR atorvastatin).

## Discussion

In this study, we tested whether statins protect from endothelial dysfunction after RT by preventing the mitochondrial injury. We established that irradiation acutely induces mitoROS production; this promotes _mt_DNA damage, which reduces transcription of mRNAs from _mt_DNA encoding ETC subunits and to lowers ETC activity; and reduced ETC activity promotes sustained mitoROS production. While both prava- and atorvastatin prevented endothelial dysfunction and cytosolic ROS, surprisingly, only pravastatin, a hydrophilic statin, reduced mitoROS production, suppressed _mt_DNA damage, and preserved activity of the ETC complex. Moreover, irradiation induced mRNA expression of the inflammatory mediators NFκB-p65, p50, and TNFα, which was prevented by pravastatin but not atorvastatin. These findings shed light on the mechanistic underpinnings of the vasoprotective effects of statins after irradiation.

In the absence of agents to specifically prevent or treat for RT-induced cardiovascular disease, aspirin, colchicine, and statins have been proposed as potential therapies (14). In a retrospective cohort study of cardiac patients post RT, statin use was associated with a significant 32% reduction in stroke, and a strong trend toward reducing the composite outcome of cardiovascular and cerebrovascular events (16). A multivariate analysis of known predictors of cardiovascular events in 1,100 patients demonstrated that statin use was associated with a reduction in both stroke and transient ischemic attacks over a median follow-up period of 3.4 years after RT (15). Of note, these studies did not provide data about which statin had been administered. Statins are particularly interesting as preventive measures because several studies have also reported anticancer properties (48, 49). In prostate and rectal cancer, statin use is associated with improved outcomes and response to chemoradiation therapy (50, 51). In a retrospective study with the SEER-Medicare dataset, patients with head-and-neck cancer taking a statin had improved overall as well as cancer-specific survival compared to those not taking a statin (52).

Although the protective effects of statins in RT-induced vascular injury have been attributed to cholesterol-lowering and anti-oxidant effects, the molecular underpinnings of the protective effects after irradiation are not fully understood. Most studies of cardioprotection by statins after irradiation have focused on inhibition of TGFβ-induced fibrotic signaling; they have not investigated specific mechanisms leading to endothelial dysfunction or other pre-atherosclerotic events.

In this study, we set out to determine the effects of statins on mitochondria. Our rationale was that mitochondria are particularly susceptible to damage by irradiation, in part because the machinery that repairs _mt_DNA is rudimentary and leaves the DNA only partially repaired. This can lead to altered expression of ETC subunits (confirmed by our study). It can also cause a chronic increase in mitoROS production, genomic instability, and epigenetic changes that are propagated upon cell division (53–55). A second reason that mitochondrial injury affects the response to irradiation is that DNA repair depends on ATP production, and this is impaired after RT (21, 54). In a recent study, we demonstrated that blocking the production of mitoROS during irradiation prevents _mt_DNA damage, loss of NO production, and endothelium-dependent dilation of the carotid artery (55); our findings from the current study are consistent with, and expand on, each of these observations and demonstrates that pravastatin prevents the deleterious effects of RT in vascular disease in part by its actions in mitochondria.

Available data on the effects of statins on mitochondrial function in various organs, notably skeletal muscle and liver, are inconsistent and partially contradictory (56, 57). Our observation that two different statins have distinct effects related to their sites of action are in line with previous reports and may provide explanations for them. Some discrepancies have been reported previously. For example, in a rat skeletal muscle cell line, lipophilic statins (such as atorvastatin) were found to be more toxic to mitochondria than the hydrophilic statin pravastatin (57). In a second example, a study in ECs confirmed that the toxic effects of 10 mM pravastatin were smaller than those of 10 mM atorvastatin (58). In pancreatic β-cells, atorvastatin, but not pravastatin, affected the mitochondrial metabolism by suppressing the antioxidant defense system and enhancing ROS production (59). Mechanisms proposed to account for these findings included decreased levels of mevalonate and of mitochondrial antioxidant coenzyme Q. Although these reports are consistent with our finding that atorvastatin leads to higher levels of mitoROS, our demonstration that pravastatin abolishes mitochondrial injury after irradiation points towards an additional protective mechanism that remains to be elucidated, including effects on mitochondrial fission and fusion.

The current study has several limitations. We focused on early time points (i.e., up to 10 days after RT) because our goal was to identify molecular pathways that are activated at the time of irradiation. Further studies are needed to ascertain whether the administration of statins at the time of RT only is sufficient to preserve endothelial function and reduce the development of atherosclerosis months to years later. Here, we investigated the effects of two specific statins, pravastatin, and atorvastatin. Further experiments are needed to understand whether differences in their effects on mitochondria are indeed attributable to group effects (i.e., their hydrophilic vs. hydrophobic natures). Moreover, the mechanistic studies were conducted *in vitro*, and the results may not be generalizable to *in vivo* conditions in patients.

In our experiments, mice and ECs were treated with concentrations of pravastatin and atorvastatin that are identical, or very similar, to those reported previously (59–61). However, we only tested a small range of concentrations and more extensive studies will be necessary to establish dose effects.

Lastly, further studies are needed to explore the implications of our findings for clinical practice.

Thus, although our findings in mice imply that pravastatin is more efficacious than atorvastatin, further studies in humans are needed to determine the extent to which our findings with different statins will translate to patients undergoing RT. Notwithstanding the need for further work as detailed here, our current data justify such studies, including a more detailed comparison of the use of specific statin types in human cohorts.

In summary, our study provides a mechanistic explanation for how the administration of statins during RT lowers the risk of developing irradiation-associated atherosclerotic vascular disease. In addition, it demonstrates that some statins, potentially because of their specific chemical properties, activate protective mechanisms in mitochondria and decrease inflammatory responses after RT.

## Data Availability

The original contributions presented in the study are included in the article/Supplementary Material, further inquiries can be directed to the corresponding author/s.
