# Corrigendum: Mechanisms by which statins protect endothelial cells from radiation-induced injury in the carotid artery

**DOI:** 10.3389/fcvm.2025.1523371

**Published:** 2025-04-11

**Authors:** Karima Ait-Aissa, Linette N. Leng, Nathanial R. Lindsey, Xutong Guo, Denise Juhr, Olha M. Koval, Isabella M. Grumbach

**Affiliations:** ^1^ Abboud Cardiovascular Research Center, Department of Internal Medicine, Carver College of Medicine, University of Iowa, Iowa City, IA, United States; ^2^ Department of Biomedical Sciences, Dental College of Medicine, Lincoln Memorial University, Knoxville, TN, United States; ^3^ Free Radical and Radiation Biology Program, Department of Radiation Oncology, Carver College of Medicine, University of Iowa, Iowa City, IA, United States; ^4^ Iowa City VA Healthcare System, Iowa City, IA, United States

**Keywords:** radiation therapy, carotid stenosis, endothelium, statin, mitochondria, prevention

A Corrigendum on Mechanisms by which statins protect endothelial cells from radiation-induced injury in the carotid artery By Ait-Aissa K, Leng LN, Lindsey NR, Guo X, Juhr D, Koval OM and Grumbach IM (2023). Front Cardiovasc Med. 10:1133315. doi: 10.3389/fcvm.2023.1133315

In the published article, there was an error in Figure 1 as published. The labeling of the subfigures 1B–H is incorrect. The corrected Figure 1 and its caption appear below.

**Figure 1:**
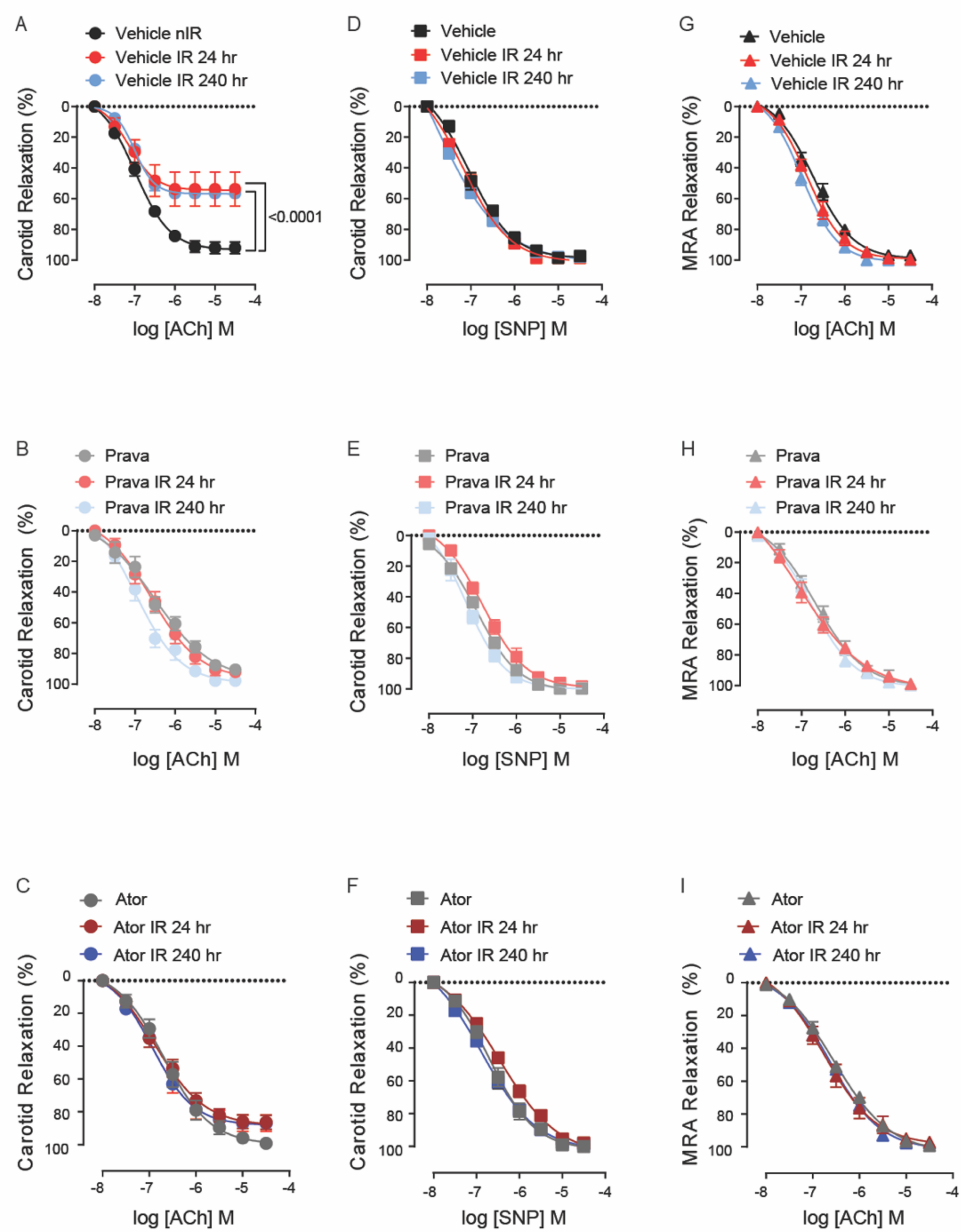
Pravastatin and atorvastatin preserve endothelial function *in vivo* following head-and-neck IR. **(A**–**C)** Effects of statins on endothelium-dependent relaxation of the carotid artery in response to acetylcholine (ACh). C57BL/6J mice were treated with **(A)** vehicle, **(B)** pravastatin (Prava), or **(C)** atorvastatin (Ator) after head-and-neck irradiation (12Gy) or sham treatment, and relaxation was tested at 24 and 240 h after irradiation. **(D**–**F)** Effects of statins on endothelium-independent relaxation of the carotid artery in response to sodium nitroprusside (SNP), in mice treated as in A, B, and C, respectively. **(G**–**I)** Effects of statins on endothelium-dependent relaxation of mesenteric resistance arteries (MRAs), in mice treated as in A, B, and C, respectively. *n* = 5 mice per group. *p* values were determined using repeated measures 2-way ANOVA followed by Tukey's *post-hoc* test.

The authors apologize for this error and state that this does not change the scientific conclusions of the article in any way. The original article has been updated.

In the published article, there was an error in Figure 3 as published. Images for Vehicle-treated non-irradiated (V-nIR) mitoSOX staining were inadvertently mismatched with those of mitotracker staining and merged images. The corrected Figure 3 and its caption appear below.

**Figure 3:**
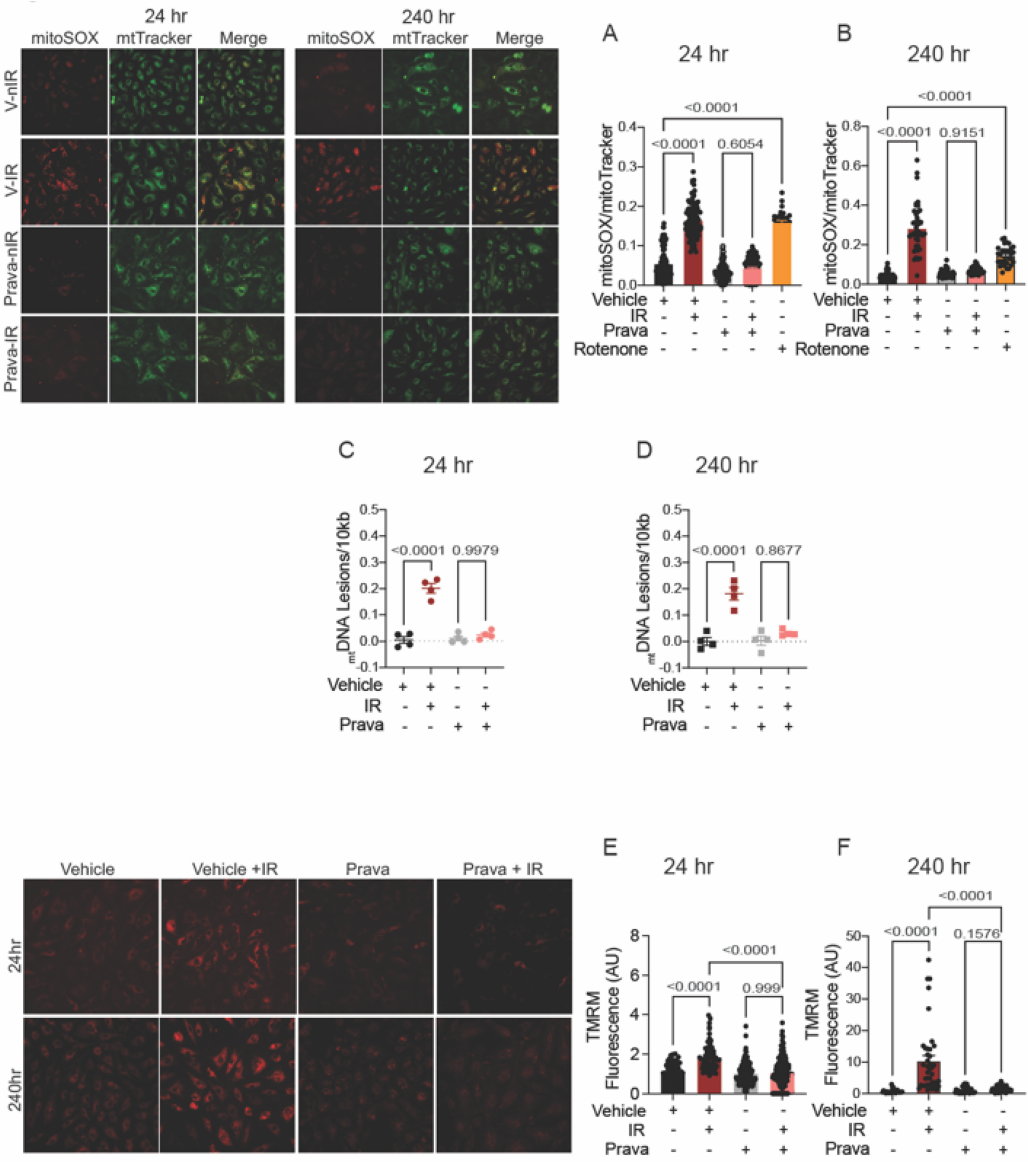
Pravastatin protects against IR-induced mitochondrial damage or hyperpolarization *in vitro*. All panels compare cells subjected to irradiation (4Gy) after pretreatment with pravastatin (Prava, 10 μM) starting at 18 h before irradiation. **(A,B)** Representative images and signal integrated density of mitoSOX fluorescence normalized to mitoTracker fluorescence in HCAECs at **(A)** 24 and **(B)** 240 h after IR. **(C,D)**_mt_DNA lesions in DNA extracted from HUVECs at **(C)** 24 h and **(D)** 240 h after IR. **(E,F)** Representative images and integrated density of mitochondrial membrane potential in HCAECs, as determined by TMRM fluorescence, at **(E)** 24 h and **(F)** 240 h after irradiation. Analysis per cell, *n* = 4 independent experiments. *p* values were determined by Kruskal–Wallis test.

The authors apologize for this error and state that this does not change the scientific conclusions of the article in any way. The original article has been updated.

In the published article, there was an error in Figure 4 as published. In Figure 4A, images for mitoSox and mtTracker staining of atorvastatin-treated cells in the left upper panels were mistakenly switched. We confirmed that the analysis was performed in the correct panels.

Figures 4C,D: The labeling for the samples treated with atorvastatin and/or IR is incorrect. The correct description for radiation treatment (IR) should be “− + − +” instead of “− + + +”. Figures 4E,F: Images for TMRM in the left lower panel are mislabeled. The correct labeling of the images should read: Vehicle, Vehicle + IR, Atorva, Atorva + IR (instead of Vehicle, Vehicle + IR, Prava, Prava + IR).

The corrected Figure 4 and its caption appear below.

**Figure 4:**
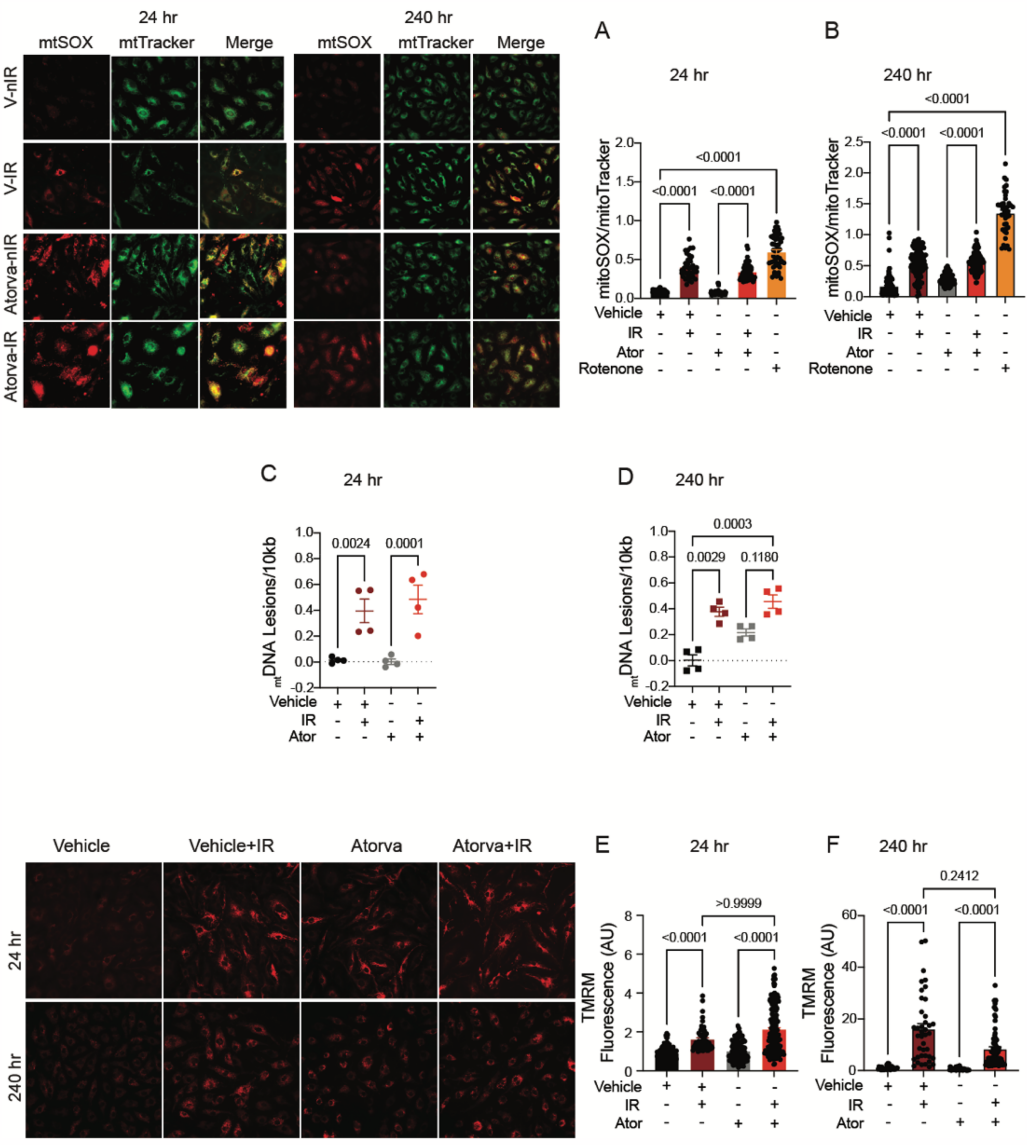
Atorvastatin does not protect against IR-induced mitochondrial damage or hyperpolarization *in vitro*. All panels compare HCAECs subjected to irradiation (4 Gy) after pretreatment with atorvastatin (Ator, 5 μM) or vehicle. Parameters assessed are: **(A,B)** Representative images and signal integrated density of MitoSOX fluorescence normalized to MitoTracker fluorescence at **(A)** 24 and **(B)** 240 h after irradiation, in cells treated with atorvastatin (5 μM) or vehicle starting 18hr before irradiation. **(C,D)** Damage to _mt_DNA as assessed by PCR assay. _mt_DNA lesions at **(C)** 24 and (**D**) 240 h after irradiation, in HUVECs treated with atorvastatin or vehicle starting 18 h before IR. **(E,F)** Representative images and integrated density of mitochondrial membrane potential, as determined by TMRM fluorescence, at **(E)** 24 and **(F)** 240 h after irradiation. Analysis per cell, *n* = 4 independent experiments, *p* values by Kruskal–Wallis test.

The authors apologize for this error and state that this does not change the scientific conclusions of the article in any way. The original article has been updated.

In the published article, there was an error in Figure 7 as published. The labeling for the samples treated with atorvastatin and/or IR in Figures 7E–H is incorrect. The correct description for radiation treatment (IR) should be “− + − +” instead of “− + + +”. The corrected Figure 7 and its caption appear below.

**Figure 7:**
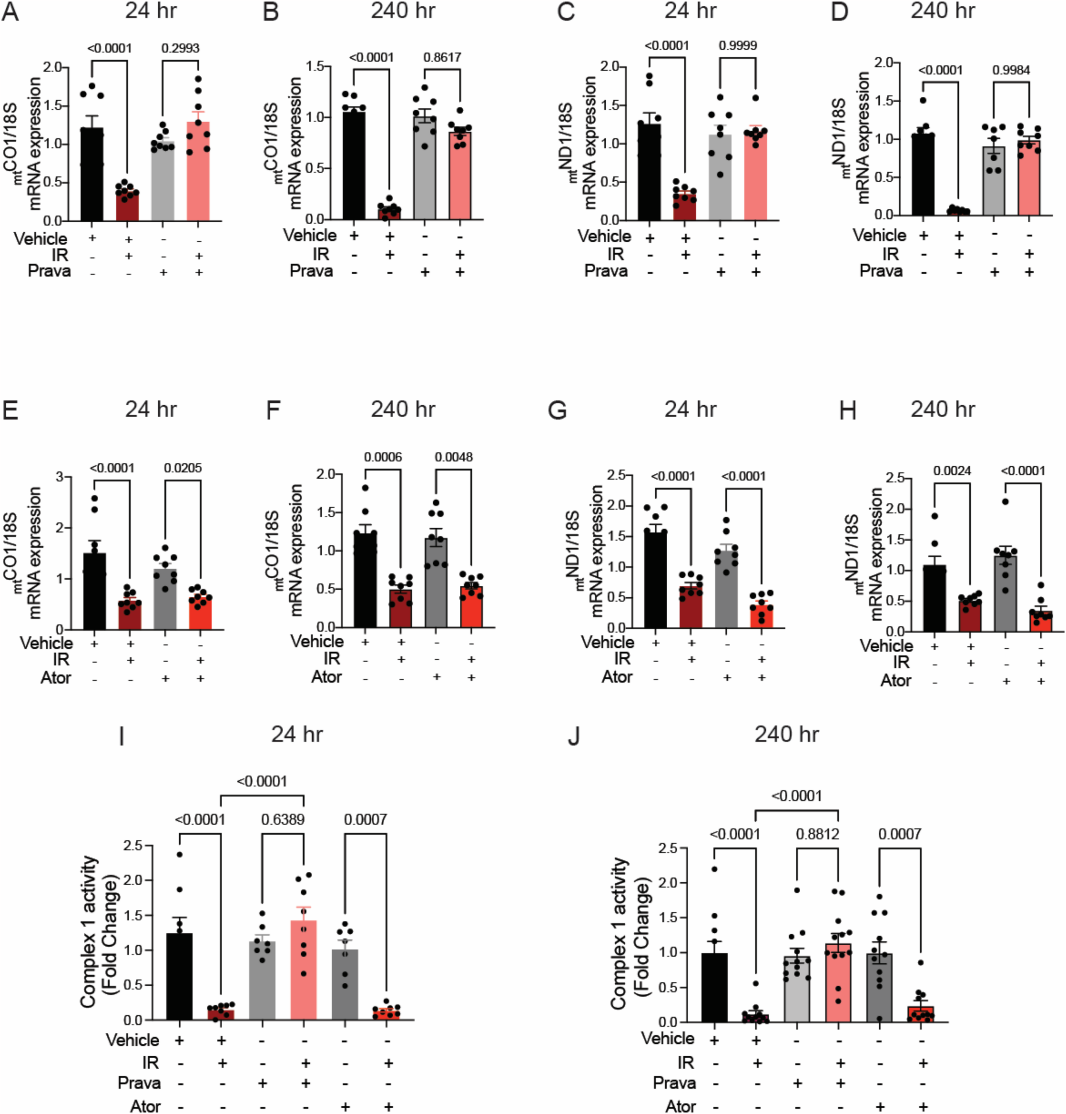
Pravastatin, but not atorvastatin, prevents irradiation-induced reduction of _mt_DNA transcription and ETC activity. All panels compare HCAECs subjected to irradiation (4Gy) after pretreatment with atorvastatin (Ator, 5 μM, overnight), pravastatin (Prava, 10 μM, overnight) or vehicle. **(A**–**D)** Effects of pretreatment with pravastatin (Prava, 10 μM, overnight) on transcriptional activity. (**A,B**) Quantitative (q)RT-PCR for cytochrome c oxidase I (MT-COI) at **(A)** 24 and **(B)** 240 h after irradiation. **(C**–**D)** qRT-PCR for NADH-ubiquinone oxidoreductase chain 1 (MT-ND1) at **(C)** 24 and **(D)** 240 h after irradiation. **(E**–**H)** Effects of pretreatment with atorvastatin (Ator, 5 μM, overnight) on transcriptional activity. (**E,F**) qRT-PCR for MT-COI at **(E)** 24 and **(F)** 240 h after irradiation. **(G,H)** qRT-PCR for MT-ND1 at **(G)** 24 and **(H)** 240 h after irradiation. (**I,J**) Activity of ETC complex 1, as assessed by fluorometric assay at **(I)** 24 and **(J)** 240 h after irradiation. *p* values by Kruskal–Wallis test.

The authors apologize for this error and state that this does not change the scientific conclusions of the article in any way. The original article has been updated.

In the published article, there was an error in Supplementary Figure 3. The labeling for the samples treated with atorvastatin and/or IR in Supplementary Figures S3E–H is incorrect. The correct description for radiation treatment (IR) should be “− + − +” instead of “− + + +”. The corrected figure and its caption appear below.

**Supplementary Figure 3:**
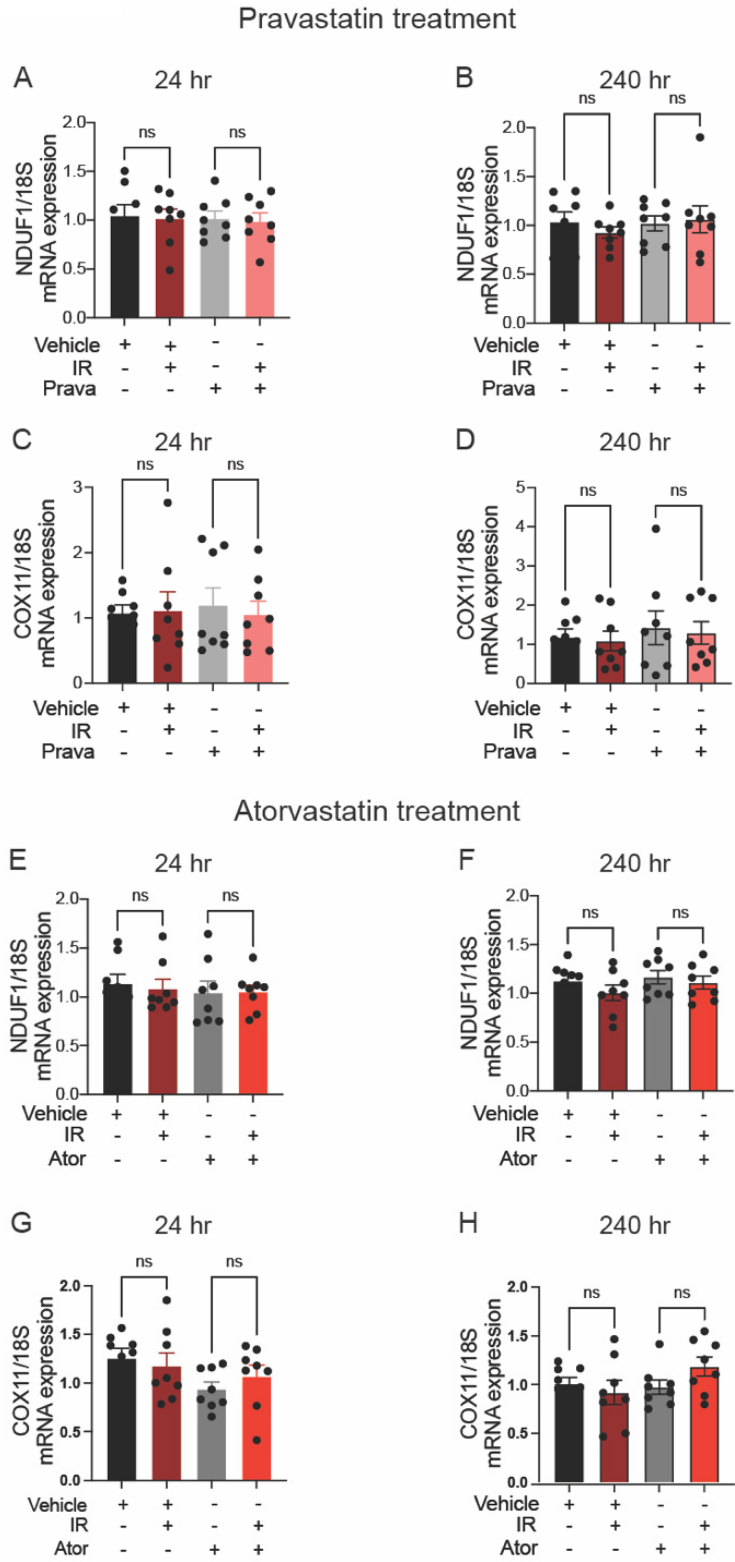
Neither pravastatin nor atorvastatin affects IR-induced transcription of nuclear DNA. **(A**–**D)** Effects of pretreatment with pravastatin (Prava, 10 μM, 1 h) on nucDNA damage in HCAECs after irradiation (IR, 4 Gy). **(A,B)** Quantitative (q)RT-PCR for NADH dehydrogenase [ubiquinone] 1 alpha subcomplex subunit 1 [**(B)**, NDUF1], with cDNA normalized to 100 ng at 24 and 240 h after IR. **(C,D)** qRT-PCR for cytochrome c oxidase 11 [**(D)**, COX11], with cDNA normalized to 100 ng at 24 and 240 h after IR. **(E**–**H)** Effects of pretreatment with atorvastatin (5 μM, overnight) on nucDNA damage in HCAECs subjected to IR. **(E,F)** qRT-PCR for NDUF1 **(B)**, with cDNA normalized to 100 ng at 24 and 240 h after IR. **(G,H)** qRT-PCR for COX11 **(D)**, with cDNA normalized to 100 ng at 24 and 240 h after IR. Statistical analysis by Kruskal–Wallis test. Ns indicates not significant.

The authors apologize for this error and state that this does not change the scientific conclusions of the article in any way. The original article has been updated.

