# Supplementary material for: Mechanisms by which statins protect endothelial cells from radiation-induced injury in the carotid artery
Source: Front Cardiovasc Med. 2023 Jun 19;10:1133315. doi: 10.3389/fcvm.2023.1133315 (PMC10315477; doi:10.3389/fcvm.2023.1133315)
Supplement: Supplementary file 1 [file Datasheet1.pdf]

Mechanisms by which statins protect endothelial cells from radiation-induced injury in the carotid artery

Karima Ait-Aissa [1,2*], Linette N. Leng [1], Nathanial R. Lindsey [1], Xutong Guo [1], Denise Juhr [1], Olha M. Koval [1], Isabella M. Grumbach. MD, PhD [1,3,4*]

[1] Abboud Cardiovascular Research Center, Department of Internal Medicine, Carver College of Medicine, University of Iowa

[2] Department of Biomedical Sciences, Dental College of Medicine, Lincoln Memorial University

[3] Free Radical and Radiation Biology Program, Department of Radiation Oncology, Carver College of Medicine, University of Iowa

[4] Iowa City VA Healthcare System, Iowa City, IA

Short title: Statins and vascular injury after radiation

Supplemental materials

Reagents:

Phenylephrine (#0754) was obtained from Amresco, acetylcholine (#A6625) and sodium nitroprusside (#S0501) were obtained from Sigma-Aldrich, and Nomega-Nitro-L-arginine (L-NNA, # ab141312) was obtained from Abcam.

Human coronary artery endothelial cells (HCAECs) were kindly provided by Drs. Gerene Denning and Lynn L Stoll (University of Iowa) (40), and human umbilical endothelial cells (HUVECs, # PCS- 100-013) were obtained from the American Type Culture Collection (ATCC). Endothelial cells were grown in endothelial cell medium (ECM) supplemented with growth factors (#1001, ScienCell).

MitoTEMPO (#SML0737) was obtained from Enzo. MitoSOX Red (#D1168), MitoTracker Green FM (#M7514), and 6-chloromethyl-2',7'-dichlorodihydrofluorescein diacetate, acetyl ester (CMH2DCFDA, # C6827 were obtained from ThermoFisher.
Tetramethylrhodamine methyl ester (TMRM, #T668) was purchased from Molecular Probes.
ECL chemiluminescent substrate was obtained from ThermoScientific (34580).
Antibodies for NFκB-p65 and for GAPDH were purchased from Cell Signaling (CST 8284S and CST 5174S).

Supplemental methods:

Cell counts

Wells of 6-well plates were seeded with 30,000 HUVECs and the cultures were grown to 80%-

90% confluency. Then, atorvastatin or pravastatin was added at a concentration of 5 μM or 10

μM. After incubation overnight (approximately 12 hours), the HUVECs were rinsed with ECM,

trypsinized and counted using an automated cell counter (Beckman Coulter).

MTT assay

HUVECs were seated in 96-well plate at a density of 1,000 cells per well and cultured for 48 h

before performing MTT toxicity assay. Cells were incubated with statins dissolved in DMSO or DMSO overnight.

The next morning, the MTT stock solution was prepared at a concentration of 5mg/ml in PBS. 10 µl of MTT stock solution with 90 µl of media was added into each well for 2 hr. Then, media were aspirated, cells rinsed with PBS and intracellular MTT formazan crystals dissolved in 50 µl DMSO. Absorbance was measured in a microtiter plate reader at a wavelength of 570 nm.

Immunoblot

Western blot analysis for NFκB-p65 was performed in cell lysates of HUVECs cells subjected to irradiation (4Gy) in the presence or absence of Pravastatin (10µM) or Atorvastatin (5µM). Briefly, the cells were harvested, lysed in RIPA buffer supplemented with proteinase and phosphatase inhibitors, and then sonicated using a sonicator. After centrifugation for 10 min at 10,000 rpm, the total protein was quantified using the BCA assay and 20 µg of protein per sample were loaded into SDS-PAGE gels. The proteins were then transferred to PVDF membranes, incubated in 5% milk and then, with primary antibodies for NFκB-p65 (1:1000) and GAPDH (1:5000) as a loading control. Blots were washed 3 times for 10 min with 0.05% Tween-20 in TBS, incubated for 1 hr at room temperature with the respective secondary antibodies, and then washed again. The blots were then developed using ECL chemiluminescent substrate according to the manufacturer’s instructions.

Supplementary table: Primer sequences used for RT-PCR

Nuclear Factor kappa B (NFκB) p50	Forward	5’-TGGACAGCAAATCCGCCCTG-3’
	Reverse	5’-TGTTGTAATGAGTCGTCATCCT-3’
NFκB p65	Forward	5′-AGGCAAGGAATAATGCTGTCCTG
	Reverse	5′-ATCATTCTCTAGTGTCTGGTTGG-3’
Tumour Necrosis Factor alpha (TNFα)	Forward	5’-CACTAAGAATTCAAACTGGGGC-3’
	Reverse	5’-GAGGAAGGCCTAAGGTCCAC-3’
Cytochrome C Oxidase I (mt- COI)	Forward	5’-TCGCAATTCCTACCGGTGTC-3’
	Reverse	5’-CGTGTAGGGTTGCAAGTCAGC-3’
NADH Ubiquinone Oxidoreductase Chain 1 (mt-ND1)	Forward	5’-GCACCTACCCTATCACTCACA-3’
	Reverse	5’-GTTTGGGCTACGGCTCG-3’
NADH Dehydrogenase [ubiquinone] 1 Alpha Subcomplex subunit 1 (NDUF1)	Forward	5’-ATGTGGTTCGAGATTCTCC-3’
	Reverse	5’-GCAACCCTTTTTTCCTTGC-3’
Cytochrome C Oxidase 11 (COX11)	Forward	5’AGGAAGAGAGTGGTGTTTTTTATTGGGTAAGTTGT3’
	Reverse	5'CAGTAATACGACTCACTATAGGGAGAAGGCTACCT TAACTACCA-AACTCCTC3’
Ribosomal 18S	Forward	5’-CCCTATCAACTTTCGATGGTAGTCG-3’
	Reverse	5’-CCAATGGATCCTCGTTAAAGGATTT-3’

Supplemental Figure 1

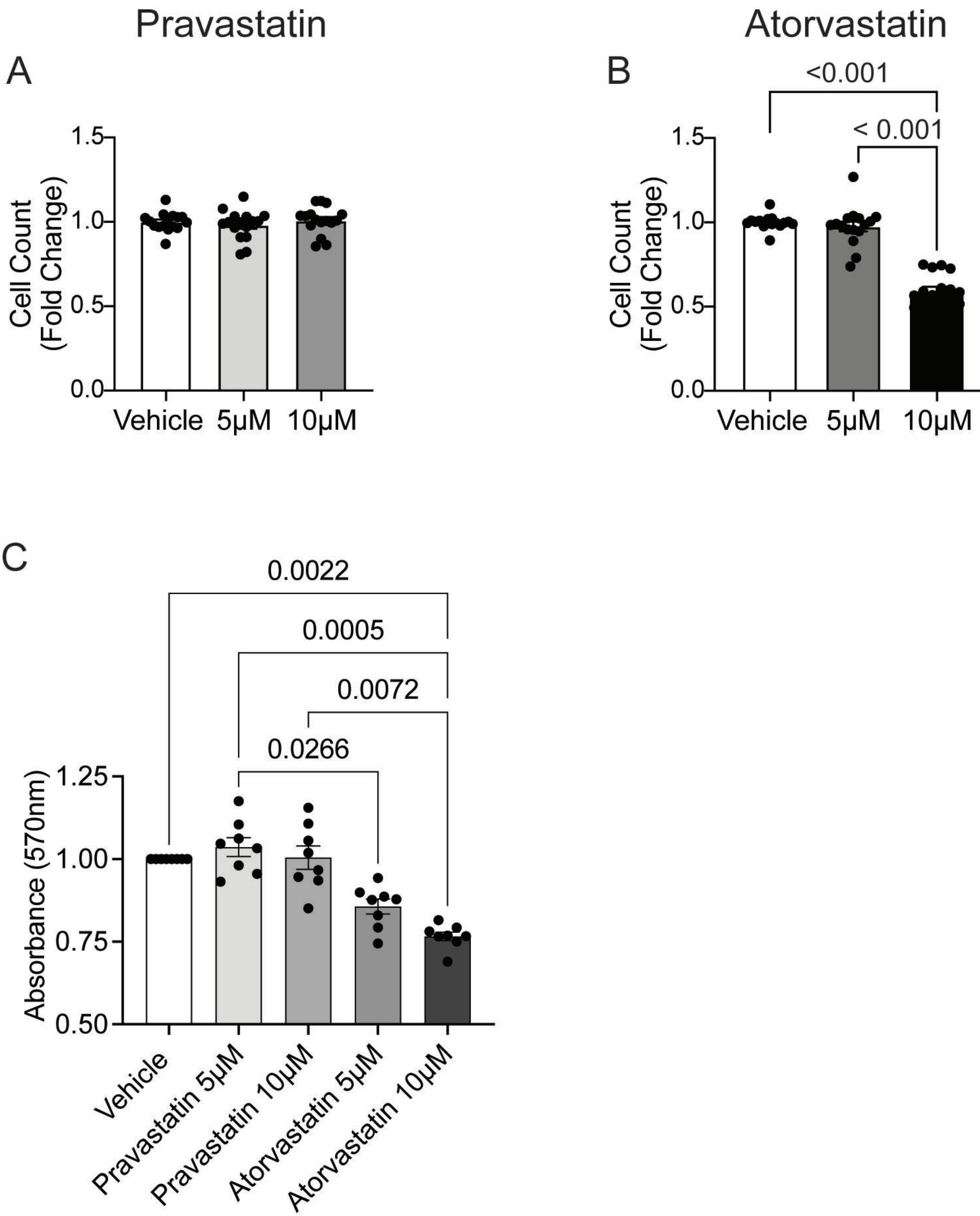

Supplemental Figure 1: Cytotoxicity assays following Pravastatin and Atorvastatin treatment.

All panels compare HCAECs subjected to pretreatment with pravastatin or atorvastatin. Cell survival measurement in the presence of pravastatin (Prava, 5 μM and 10 μM, overnight) (**A**) or Atorvastatin (Atorva, 5 μM and 10 μM, overnight) (**B**). Cell viability measurement via MTT assay in cells pretreated with pravastatin or atorvastatin (5 μM and 10 μM, overnight)(**C**). Statistical analysis by Kruskal-Wallis test.

Supplemental Figure 2

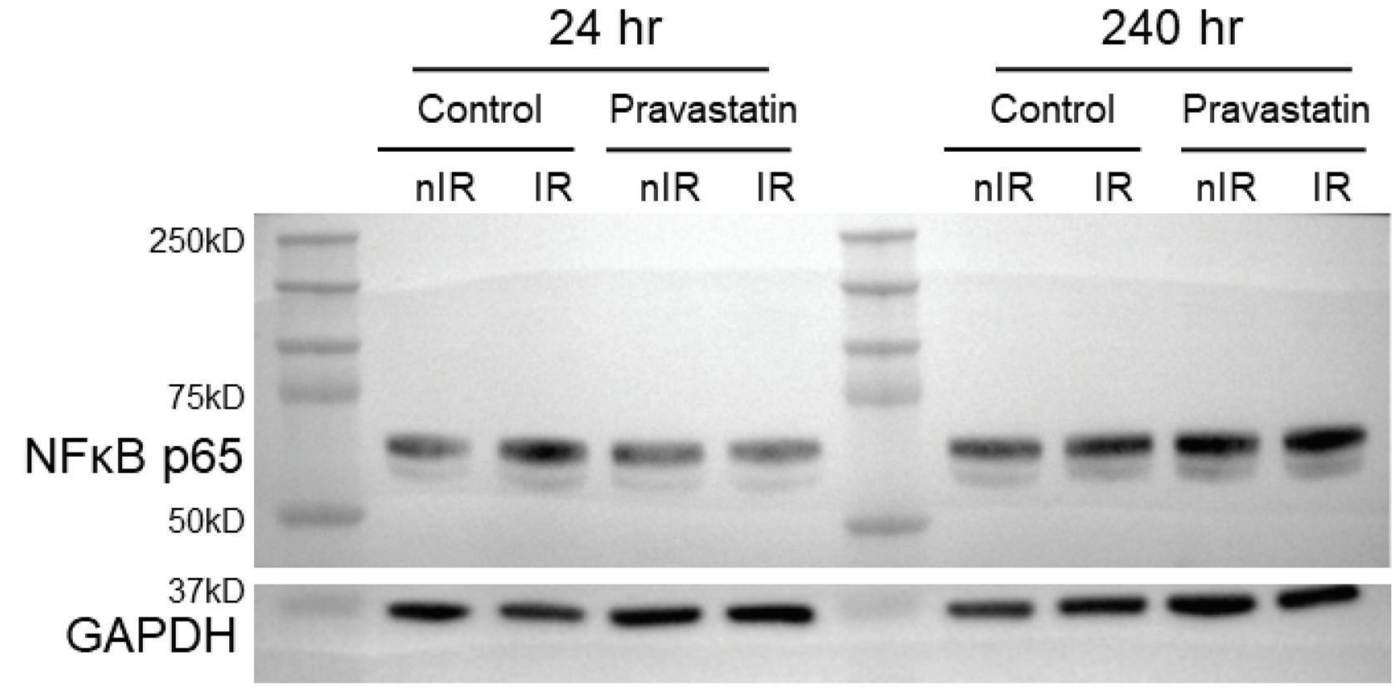

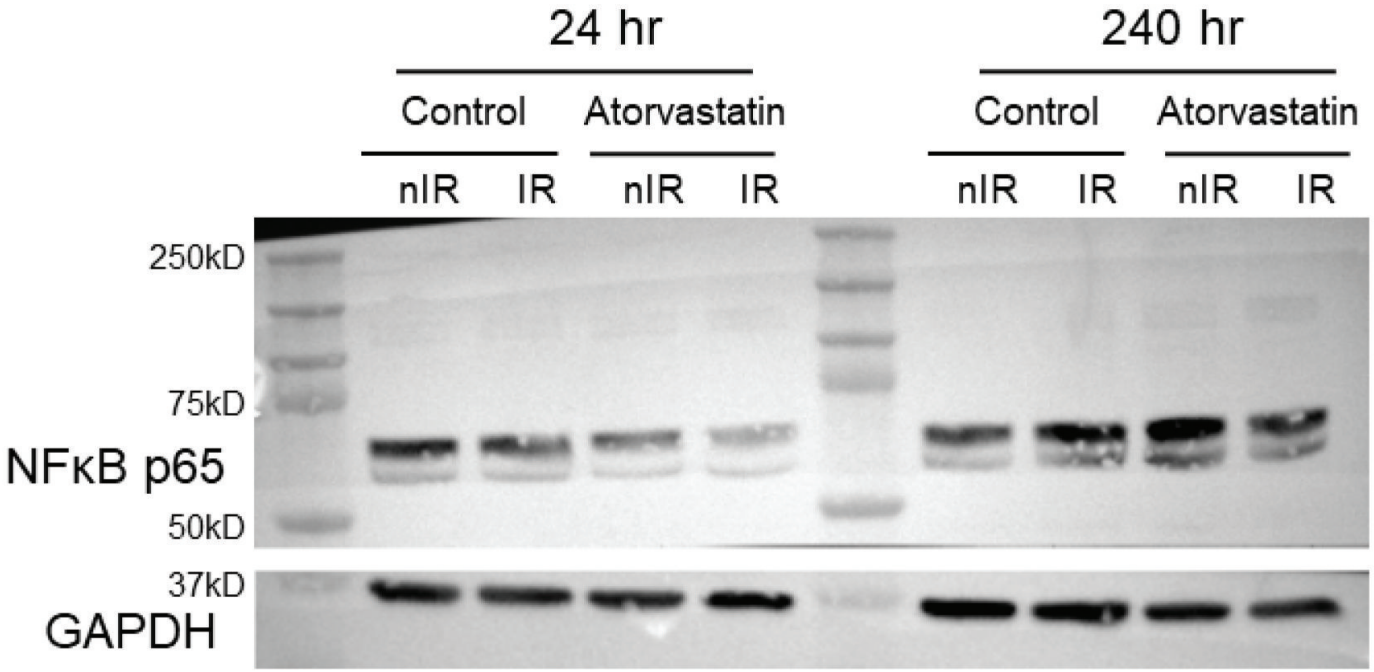

Supplemental Figure 2: Protein expression of NFkB-p65 following irradiation in endothelial cells.

Western blot analysis for NFkB-p65 and GAPDH from lysates of HUVECs cells subjected to irradiation (4Gy) in the presence of pravastatin (10uM) or atorvastatin (5uM) at 24 hr and 240 hr.

Supplemental Figure 3

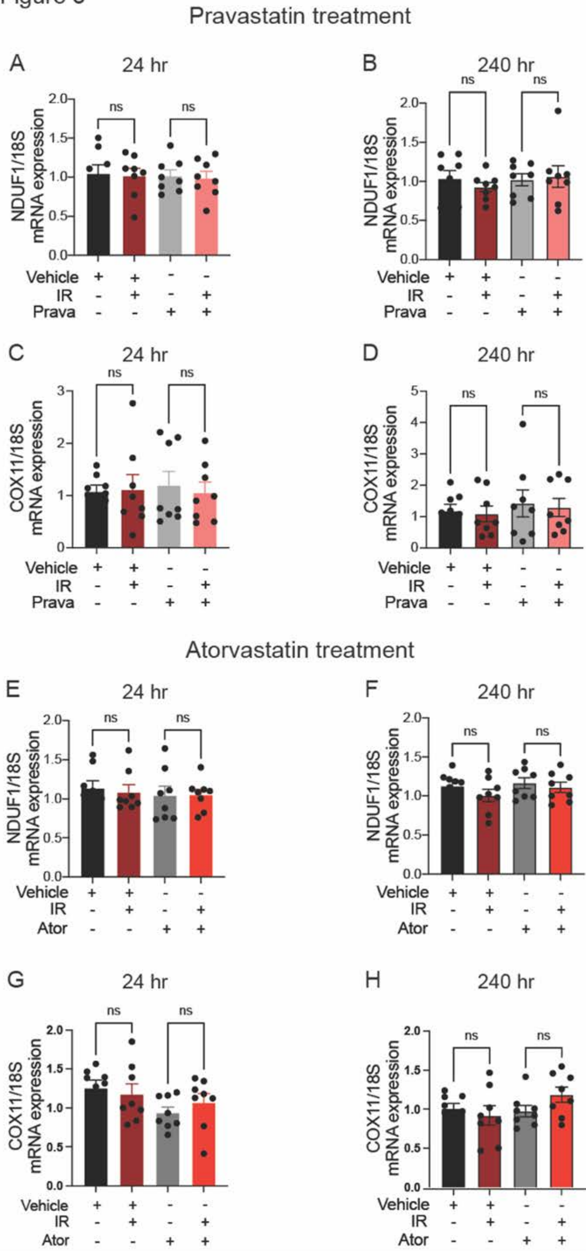

Supplemental Figure 3: Neither pravastatin nor atorvastatin affects IR-induced transcription of nuclear DNA.

(A-D) Effects of pretreatment with pravastatin (Prava, 10 μM, 1 hr) on nucDNA damage in HCAECs after irradiation (IR, 4 Gy). (A, B) Quantitative (q)RT-PCR for NADH dehydrogenase [ubiquinone] 1 alpha subcomplex subunit 1 (B, NDUF1), with cDNA normalized to 100 ng at 24 and 240 hr after IR. (C, D) qRT-PCR for cytochrome c oxidase 11 (D, COX11), with cDNA normalized to 100 ng at 24 and 240 hr after IR. (E-H) Effects of pretreatment with atorvastatin (5 μM, overnight) on nucDNA damage in HCAECs subjected to IR. (E, F) qRT-PCR for NDUF1 (B), with cDNA normalized to 100 ng at 24 and 240 hr after IR. (G, H) qRT-PCR for COX11 (D), with cDNA normalized to 100 ng at 24 and 240 hr after IR. Statistical analysis by Kruskal-Wallis test. Ns indicates not significant.
